# Genomic insights into the origin, domestication and genetic basis of agronomic traits of castor bean

**DOI:** 10.1186/s13059-021-02333-y

**Published:** 2021-04-20

**Authors:** Wei Xu, Di Wu, Tianquan Yang, Chao Sun, Zaiqing Wang, Bing Han, Shibo Wu, Anmin Yu, Mark A. Chapman, Sammy Muraguri, Qing Tan, Wenbo Wang, Zhigui Bao, Aizhong Liu, De-Zhu Li

**Affiliations:** 1 Department of Economic Plants and Biotechnology, Yunnan Key Laboratory for Wild Plant Resources, Kunming Institute of Botany, Chinese Academy of Sciences, Kunming, 650201 China; 2 Key Laboratory for Forest Resource Conservation and Utilization in the Southwest Mountains of China, Ministry of Education, Southwest Forestry University, Kunming, 650224 China; 3 Biological Sciences and Centre for Underutilised Crops, University of Southampton, Southampton, SO17 1BJ UK; 4 Shanghai OE Biotech Co., Ltd, Shanghai, 201114 China; 5 Germplasm Bank of Wild Species, Kunming Institute of Botany, Chinese Academy of Sciences, Kunming, 650201 China

**Keywords:** Genomic evolution, Domestication, Population genetics, GWAS, Castor bean

## Abstract

**Background:**

Castor bean (*Ricinus communis* L.) is an important oil crop, which belongs to the Euphorbiaceae family. The seed oil of castor bean is currently the only commercial source of ricinoleic acid that can be used for producing about 2000 industrial products. However, it remains largely unknown regarding the origin, domestication, and the genetic basis of key traits of castor bean.

**Results:**

Here we perform a de novo chromosome-level genome assembly of the wild progenitor of castor bean. By resequencing and analyzing 505 worldwide accessions, we reveal that the accessions from East Africa are the extant wild progenitors of castor bean, and the domestication occurs ~ 3200 years ago. We demonstrate that significant genetic differentiation between wild populations in Kenya and Ethiopia is associated with past climate fluctuation in the Turkana depression ~ 7000 years ago. This dramatic change in climate may have caused the genetic bottleneck in wild castor bean populations. By a genome-wide association study, combined with quantitative trait locus analysis, we identify important candidate genes associated with plant architecture and seed size.

**Conclusions:**

This study provides novel insights of domestication and genome evolution of castor bean, which facilitates genomics-based breeding of this important oilseed crop and potentially other tree-like crops in future.

## Background

Castor bean (*Ricinus communis* L., Euphorbiaceae, 2n = 20) is an important non-food oilseed crop worldwide, with a unique seed oil profile, rich in ricinoleic acid (12-hydroxyoleic acid, 18C:1OH), which has been used in industry for making lubricants, cosmetic, coatings, inks, plastics, and biodiesel [1, 2]. In 2018, the global trade in castor oil reached $1340 million (https://www.zionmarketresearch.com/report/castor-oil-market). Its seeds contain the extremely toxic protein ricin that has been used as an immunotoxin for therapeutic purposes in different cancers, was likely used by early hunters, and has been reportedly used as a weapon [3]. In addition, castor bean seeds contain a large endosperm that is persistent throughout seed development [4], leading to castor bean being considered a valuable model system for studying seed biology among dicots [5, 6].

Prehistoric uses of castor bean have been revealed by archeological discovery in South Africa (dated to ~ 24,000 years before present, YBP) [7] and early management has been found in Sudan (~ 7000 YBP) [8], Egypt (~ 4000 YBP) [9], and Iraq (~ 6000 YBP) [10]. These anthropological records highlight how non-food plants have been widely used by humans since prehistoric times. Presently, due to its economic importance and ease of growth in unfavorable environments, domesticated castor bean is cultivated in many regions (in particular in India, Brazil, and China) and feral plants escaped from cultivation grow worldwide. Based on morphological variation, four centers of diversity have been proposed [9, 11], comprising (i) East Africa (Kenya and Ethiopia), (ii) West Asia (Iraq, Iran, Syria, Turkey, and Afghanistan) and the Arabian Peninsula, (iii) India, and (iv) China. Since the germplasm distributed in East Africa exhibit a tree-like phenotype, with a single elongated trunk, dehiscent capsule, and small seeds, these have been suggested to represent the wild relatives of domesticated castor bean [9, 12], but this supposition lacks supporting evidence. In addition, worldwide studies revealed low genetic diversity in cultivated and landrace castor bean [13–15], which has long been thought to exacerbate the challenge of breeding in the future. It is largely unknown whether this low genetic diversity stems from genetic bottlenecks during castor bean domestication. If so, one would expect that wild relatives of castor bean contain the most diverse germplasm with rich genetic variation. However, this has not been explored to date owing to the limited availability of wild germplasm. Sampling wild castor bean would not only facilitate an understanding of the domestication, evolution, and population demographic history, but also help reappraise genome-wide genetic diversity and identify candidate genes related to key agronomic traits. Although a few studies investigating the genetic diversity of castor bean have included a few wild accessions collected from East Africa concluding that wild germplasm does indeed harbor higher genetic diversity [16, 17], the population demographic history of wild castor bean, genetic bottlenecks, selection signatures during domestication, and the genetic basis of key agronomic traits remain largely unexplored.

During broad field surveys in Kenya and Ethiopia, we therefore collected castor bean accessions with traits typical of the wild progenitor, such as dehiscent capsule, small seeds, and a tree phenotype with a single elongated trunk (Additional file 1: Fig. S1). In contrast, most cultivars and landraces are annual and dwarfed crops. In this study, we first de novo assembled a chromosome-scale genome for a wild castor bean accession and then analyzed resequencing data from 505 accessions from throughout the world. Our aim was to (i) quantify genomic variation and population structure; (ii) investigate the origin, domestication, and population demographic history; and (iii) reveal the genetic basis of plant architecture and yield-associated traits differentiating wild and domesticated castor bean. The results not only shed light on castor bean evolution, but also facilitate future genomics-assisted breeding of this important oilseed crop and potentially other tree-like crops.

## Results

### A newly assembled castor bean genome reveals its evolutionary context in the Euphorbiaceae

**Fig. 1:**
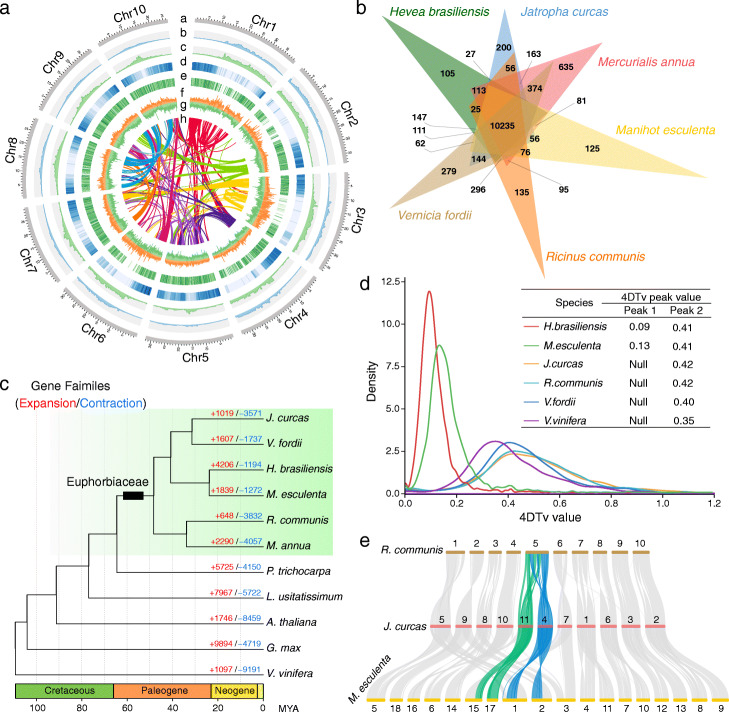
Genome assembly of wild castor bean and evolutionary analyses in the Euphorbiaceae. **a** Landscape of genomic features and genetic diversity of castor bean. Circles represent, from outermost to innermost, (a) pseudochromosomes, (b, c) DNA transposon and retrotransposon density, (d, e) the distribution of gene and their expression, (f, g) the distribution of SNPs and INDELs, and (h) intra-genome collinear blocks. **b** Orthologous gene families among six species of the Euphorbiaceae identified by OrthoFinder2. The number represents gene families identified for each species. **c** Phylogenetic tree of castor bean (*Ricinus communis*) and ten other eudicot species. The number on each branch indicates the number of genes in expanded/contracted gene families in each plant species. The black box indicates the base of the Euphorbiaceae. **d** Density of 4DTv distances for paralogous genes within six plant genomes. The peak values are shown in insets. **e** Genomic collinearity between *Ricinus communis*, *Jatropha curcas*, and *Manihot esculenta*. The highlighted lines (green and blue) indicate the collinear relationship of castor bean chromosome 5

We selected a wild castor bean tree (accession Rc039) from Ethiopia for genome sequencing (Additional file 1: Fig. S1). Based on long read sequencing using PacBio Sequel platform (~ 36.5 Gb, 102-fold genome coverage) and Hi-C sequencing technology (~ 49.2 Gb), we de novo assembled a chromosome-scale genome (~ 336 Mb) with contig N50 of 11.59 Mb and scaffold N50 of 32.06 Mb (Table 1 and Fig. 1a), consistent with the estimated genome size of ~ 356 Mb determined by the *k-mer* method based on 36.4 Gb Illumina data and from flow cytometry (Additional file 1: Fig. S2). Approximately 97.4% (~ 328 Mb) of the genome was anchored onto 10 pseudochromosomes, which was further validated by a physical map we constructed in this study (Fig. 1a and Additional file 1: Fig. S3). The BUSCO analysis revealed 2079 (98%) complete BUSCOs, 29 (1.4%) of which were duplicated (Additional file 2: Table S1). These results indicate that the newly assembled genome is complete, of high quality, and more contiguous than the previous castor bean assembly (of a cultivar named “Hale”, N50 = 0.56 Mb) [18].

**Table 1:** Summary of assembly and annotation of the wild castor bean genome

Genome assembly	
Genome size	336 Mb
N50 of contig	11.59 Mb
N50 of scaffold	32.06 Mb
GC content	33.21%
Chromosome number	10
Genome completeness (complete BUSCOs)	98%
Number of genes	25,814
Percentage of repetitive sequence	53.89%
Number of noncoding RNAs	3180

Approximately 53.9% of the wild castor bean genome is composed of repetitive elements (Table 1), comparable to that in inbred “Hale” (52.2% of the genome) [18]. Long terminal repeat (LTR) retrotransposons were the most abundant, making up 26.02% of the genome, with LTR/Gypsy elements making up more than half of these (14.4% of the genome; Fig. 1a and Additional file 2: Table S2). In total, we predicted 25,814 protein-coding genes, 40,954 transcripts, and 3180 noncoding RNAs in the Rc039 genome (Table 1 and Additional file 2: Table S3). The vast majority of gene models (~ 96.7%) received an annotation edit distance [19] score ≤ 0.5, suggesting a highly credible gene model (Additional file 1: Fig. S4). Over 92% of the predicted genes showed homology to genes with known functional annotation in a public database (Additional file 2: Table S4).

Genes in the genome were grouped into 14,206 orthogroups, and 135 orthogroups containing 291 genes were identified as castor bean-specific relative to five other members of the Euphorbiaceae (Fig. 1b and Additional file 2: Table S5). A comparison among 11 eudicot species revealed that 648 orthogroups have undergone expansion events and 3832 undergone contraction events in the Rc039 genome (Fig. 1c). These expanded orthogroups were significantly enriched (adjusted *P* < 0.05) in diverse biological processes (including photosynthesis and oxidative phosphorylation), pathways (including lysine, carotenoid, and sesquiterpenoid and triterpenoid biosynthesis), and some metabolites (including pyrimidine, propanoate, purine, linoleic acid, and glyoxylate and dicarboxylate metabolism) (Additional file 2: Table S6). Phylogenetic analysis revealed that the Euphorbiaceae and Salicaceae (represented by *Populus*) diverged ~ 64.55 million years ago (MYA). Castor bean and *Mercurialis annua* clustered together, both members of subfamily Acalyphoideae, and diverged from four other members of Euphorbiaceae (subfamily Crotonoideae) ~ 48.28 MYA (Fig. 1c), consistent with previous reports [20, 21]. Both Ks (synonymous substitution rate) and 4DTv (fourfold synonymous third codon transversion) analyses reveal that *V. fordii*, *J. curcus*, and castor bean share the ancient whole-genome triplication (γ) with *Vitis*, while for *H. brasiliensis* and *M. esculenta* experienced a recent species-specific duplication (Fig. 1d and Additional file 1: Fig. S5). Analysis of genome collinearity between castor bean (2n = 20), *J. curcus* (2n = 22), and *M. esculenta* (2n = 36) revealed a substantial degree of collinearity and several large collinear regions (Fig. 1e). Nine of the ten castor bean chromosomes are approximately collinear with those in *J. curcus*, with the exception of castor bean chromosome 5 which maps to *J. curcus* chromosomes 4 and 11. Most of the chromosomes exhibit a 1:2 projection ratio between castor bean and *M. esculenta* except for chromosome 5 with a 1:4 projection. Our analysis clearly shows how the genomes of members of the major clades of the Euphorbiaceae have diverged and duplicated (Fig. 1e).

### Population genome resequencing and genetic structure analyses prove East African accessions are the extant wild progenitors of castor bean

**Fig. 2:**
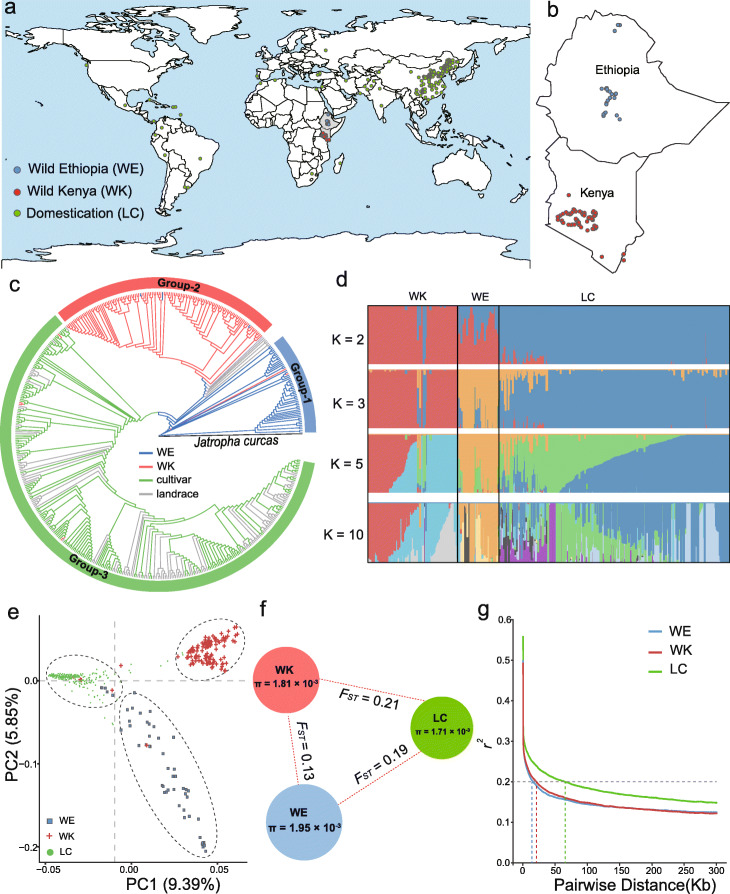
Population genomic analyses in castor bean. **a** Geographic distribution of 505 castor bean accessions. Blue, red, and green dots on the world map represent the wild Ethiopia (WE), wild Kenya (WK), and domesticated accessions (including landraces and cultivars, LC), respectively. **b** Geographic distributions of wild castor bean collected from Ethiopia (blue dots) and Kenya (red dots). **c** Phylogenetic tree of all accessions inferred from whole-genome SNPs, with *Jatropha curcas* as an outgroup. Three major clades are indicated. The line colors indicate groups of castor bean accessions (WE, WK, landrace, and cultivar). **d** ADMIXTURE plot for all castor bean accessions. The values of *K* represent the number of clusters. **e** PCA plot of the first two eigenvectors of castor bean accessions. **f** Genetic diversity (*θ*_*π*_) and divergence (*F*_*ST*_) among three castor bean populations WE, WK, and LC. **g** LD decay for three populations (WE, WK, and LC)

A total of 505 accessions including 56 wild accessions from Ethiopia (WE population), 126 wild accessions from Kenya (WK population), and 323 domesticated accessions from the world (172 landraces and 151 cultivars, LC population) were used for subsequent analysis, which covers the worldwide distribution and phenotypic diversity of castor bean (Fig. 2a, b and Additional file 2: Table S7). Of them, 280 were sequenced for this study and 225 were generated by Fan et al. [16]. On average, 97% of the clean reads were aligned onto the Rc039 genome, with an average depth of 19.5 × and coverage of 96.5% (Additional file 2: Table S8). We detected a total of 3,569,884 SNPs and 382,570 indels, equating to 10.6 SNPs and 1.14 indels per kilobase (Fig. 1a and Additional file 2: Table S9). The accuracy of SNP calls was estimated by comparing the SNPs identified from previous RNA-seq data from two accessions and genome resequencing data in two individual lines (Rc249 and Rc250; 8540 SNPs) [17, 22] with genome resequencing data. We found that 8404 RNA-seq SNPs (~ 98.4%) were detected in this study. 82.2% of the genome-wide SNPs (2,934,934) were located in intergenic regions, and 17.8% (634,950) were located in genic region. Among the latter, we observed 388,392 intronic SNPs, 149,896 exonic SNPs, and 96,662 UTR SNPs (Additional file 2: Table S9). Within the exonic regions, we annotated 83,664 non-synonymous SNPs, 51,430 synonymous SNPs, and 14,802 SNPs causing the change of predicted stop or start codons.

Subsequently, we constructed a rooted phylogenetic tree with *Jatropha curcas* as an outgroup, revealing that the 505 castor bean accessions were divided approximately into three main groups. Group 1 mainly consisted of WE accessions (53 WE and two WK), group 2 mainly consisted of WK accessions (120 WK and three WE), and group 3 mainly consisted of LC accessions (316 LC and four WK) with significant mixture of landraces and cultivars (Fig. 2c). We refer to these from hereon as WE, WK, and LC groups, respectively. We found that WE are the earliest diverging among the three groups (Fig. 2c and Additional file 1: Fig. S6), and WE and WK are divergent from the LC group suggesting WE and WK represent the extant wild progenitors of castor bean. Seven landraces that were mainly provided by USDA-Agricultural Research Center clustered with the wild populations, possibly resulting from a recent introduction from East Africa or have resulted from recent breeding (Fig. 2c and Additional file 1: Fig. S6). The WK population formed two subgroups we term WK-I and WK-II which are distributed geographically (see below; Additional file 1: Fig. S6). The LC population formed a monophyletic clade and had no distinct geographically based pattern (Additional file 1: Fig. S6) consistent with previous reports [13–15, 17]. Admixture analysis of population structure (Fig. 2d) supports the classification of groups or subgroups and backs up our inferences on the domestication history. More specifically, at *K* = 2, LC is separated from the WE and WK (wild) populations, with evidence of mixed ancestry in the WE group, and at *K* = 3, the WE, WK, and LC groups are apparent. At *K* = 5, further subgroups emerge, including the split between WK-I and WK-II, but no geographically structured subgroups were observed with the increase of *K* value, although *K* = 10 was optimal (Fig. 2d and Additional file 1: Fig. S7). The principal component analysis (PCA; Fig. 2e) revealed a similar population structure.

Associated with castor bean domestication, we observed a significant reduction of genome-wide diversity in the LC population (*θ*_*π*_ = 1.71 × 10^−3^) relative to WE (1.95 × 10^−3^) and WK (1.81 × 10^−3^) (*P* < 0.01 by Kruskal-Wallis test; Fig. 2f and Additional file 1: Fig. S8a), consistent with the general pattern of wild populations harboring higher genetic diversity than domesticated populations. However, this ratio of diversity (*π*_wild_/*π*_cultivar_) in castor bean (1.14) was quite small relative to other crops such as rice (1.25), soybean (1.58), cucumber (1.96), and tomato (2.63) [23] suggesting an overall weak domestication bottleneck. Pairwise *F*_*ST*_ between populations indicates obvious genetic divergence between wild and domesticated population (WE and LC: *F*_*ST*_ = 0.19, WK and LC: *F*_*ST*_ = 0.21) but less between the WE and WK populations (*F*_*ST*_ = 0.13, Fig. 2f). Decay of linkage disequilibrium (LD) occurred over a substantially shorter distance in wild populations (~ 15.3 kb for WE and ~ 20.8 kb for WK to decay to *r*^*2*^ = 0.2) than in the domesticated population (~ 64.5 kb for LC) (Fig. 2g), correlating with expectations based on greater outcrossing in wild castor bean than domesticates [14].

Four centers of phenotypic diversity have been proposed [9, 11]; therefore, we estimated the genetic diversity for the three geographic groups in Asia including West Asia (including Turkey, Syria, Iraq, and Iran), South Asia (Pakistan and India), and China and compare those to the fourth center in East Africa. West Asian castor bean harbored relatively high nucleotide diversity (*θ*_*π*_ = 1.90 × 10^−3^) comparable to the wild group and substantially greater than found in South Asian and Chinese groups (1.66 × 10^−3^ and 1.56 × 10^−3^, respectively). The potential reason for high genetic diversity in this area is that accessions in West Asia may have repeatedly received gene flow from wild castor bean in East Africa or that this represents the earliest group of domesticates, with other Asian accessions being founded from this region. We employed TreeMix to measure gene flow and migration and found, indeed, that gene flow from Ethiopia to West Asia was supported (weight = 0.24, Additional file 1: Fig. S9). While it is possible for wild castor bean to have previously existed in this area, archeological remains of only cultivated castor bean seeds have been reported in this region (from Iraq dating to ~ 6000–7000 YBP [10]). Pairwise *F*_*ST*_ shows low differentiation between Chinese and Indian castor bean (*F*_*ST*_ = 0.09) and greater differentiation between these Eastern sites and West Asian castor bean (*F*_*ST*_ = 0.19 between Chinese and West Asian accessions and *F*_*ST*_ = 0.11 between India and West Asian accessions).

Taken alongside archeological evidence, our results clearly show that accessions from East Africa are the extant wild progenitors of castor bean and that domestication occurred somewhere between East Africa and West Asia, and these are the main centers of diversity. Following this, accessions were distributed throughout the world. The lack of geographically structured genomic variation in the landraces and cultivars suggests continued and multi-directional transport and/or breeding of castor bean.

### Population demographic history reveals genetic bottleneck and vicariance

**Fig. 3:**
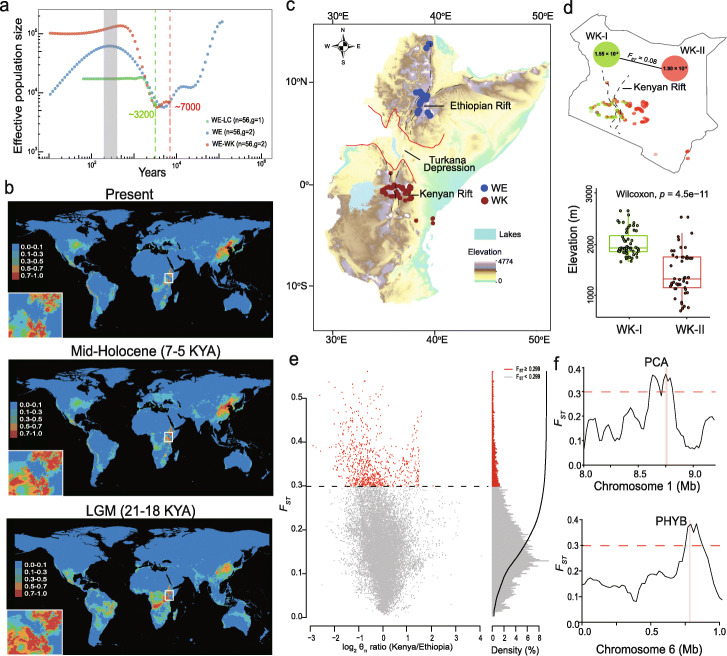
Population demographic history and genetic differentiation of castor bean. **a** Effective population size and split time inferred by SMC++ based on WGS SNPs for WE (blue dots), WK (red dots), and LC (green dots). The red and green dash lines indicate the split time for WE/WK and WE/LC, respectively. The gray bar indicates the maximum of effective population size ~ 400–200 years before present (YBP) after a population bottleneck (6000–4400 YBP). **b** Predicated distributions of castor bean based on ecological niche modeling. Areas in different colors indicate the various probabilities (0–1; blue to red) of suitable habitats for castor bean. LGM last glacial maximum, KYA thousand years ago. **c** Map of the East Africa Rift system including Turkana Depression (between red lines) and Ethiopian and Kenyan rifts (dashed black line). Lakes and elevation are indicated. Collection sites of accessions are indicated. **d** (Top) Nucleotide diversity and genetic divergence between the two genetic groups WK-I and WK-II and their geographic locations, (bottom) elevational distributions of the two genetic groups WK-I and WK-II. **e** Genetic divergence between WE and WK populations. Red dots indicate the regions that scored in the top 5% of *F*_*ST*_ (dashed line shows cut-off). **f** Two highly divergent regions between WE and WK populations. Candidate genes *PCA* and *PHYB* with the top 5% of *F*_*ST*_ are shown. The dashed horizontal line indicates the threshold for the top 5% of *F*_*ST*_ (0.299)

To better understand the demographic history of castor bean, we employed the SMC++ method to infer effective population size (*N*_*e*_) through time and divergence times between castor bean populations. We used unphased SNPs and a mutation rate of 6.9 × 10^−9^ mutations per nucleotide per year (see the “Methods” section). We found that castor bean underwent a continual reduction in *N*_*e*_ between 100,000 YBP and 6000–4400 YBP from ~ 80,000 to ~ 7400 in the LC population and to 2800 in the WE and WK populations (Fig. 3a and Additional file 1: Fig. S10), suggesting a severe population bottleneck in all castor bean populations. After this bottleneck, we observed a gradual increase in *N*_*e*_ reaching a maximum 400–200 years ago (Fig. 3a and Additional file 1: Fig. S10), which could be linked to increasing cultivation of castor bean worldwide for emerging industry applications of castor oils at that time [9]. The LC population separated from wild East African castor bean ~ 3200 YBP (Fig. 3a), consistent with the archeological record from Egypt indicating the cultivation of castor bean could be dated back to 3000–4000 YBP [9].

For wild castor bean, we estimate that divergence between WK and WE occurred ~ 7000 YBP, roughly coinciding with the reduction of *N*_*e*_ occurring ~ 6000 YBP (Fig. 3a and Additional file 1: Fig. S10). Ecological niche modeling (ENM) revealed an obvious reduction or even disappearance of potential castor bean habitats during the Mid-Holocene (7000–5000 YBP) in the Turkana Depression (TD) (Fig. 3b), a topographic corridor within the East African Rift System, which interrupts the connection between northern Kenya and southern Ethiopia (Fig. 3c) [24]. Estimating the relative contribution of environmental factors used to the potential distribution pattern of castor bean worldwide reveals that mean annual temperature, followed by precipitation, are the most significant climate variables (Additional file 2: Table S10). Taken together, we speculate that genetic differentiation between WK and WE wild castor bean is likely related to climate change in the TD during the Mid-Holocene. Accumulating evidence suggests that dramatic climate change in the TD, especially extreme aridity, frequently occurred during this period [25], which had considerable effects on human migration, disappearance of vegetation cover, and sharp declines of lake water levels [25, 26]. In addition, we found that genetic diversity was significantly higher in WK-II than WK-I (*θ*_*π*_ = 1.80 × 10^−3^ and *θ*_*π*_ = 1.55 × 10^−3^, respectively). Despite low genetic divergence between these two subgroups (*F*_*ST*_ = 0.08; Fig. 3d and Additional file 1: Fig. S8b), the two groups show distinct geographical locations on either side of the Kenyan Rift and are found at significantly different elevations (Fig. 3d). This indicates that the contemporary environment and recent climatic change associated with the East Africa rift system have had a substantial influence on the genetic diversity and differentiation of wild progenitors of castor bean, and potentially other species.

To explore differentiation that may be involved in local adaptation of the WK and WE populations, we examined *F*_*ST*_ throughout the genome to identify divergent regions using sliding 20-kb windows, roughly corresponding to the distance of LD decline. In total, we identified 808 highly divergent regions (~ 29.9 Mb) that scored in the top 5% of the distribution of *F*_*ST*_ (*F*_*ST*_ > 0.299), encompassing 2647 genes (Fig. 3e and Additional file 2: Table S11). Although these genes were not significantly enriched in specific GO terms, many genes involved in the establishment of localization, reproductive process, response to stimulus, and regulation of biological process were identified (Additional file 2: Table S12). For example, we identified the Rc01G001244 encoding a putative FCA protein known to regulate flowering time and thermal adaptation in *Arabidopsis* [27, 28]; Rc01G002857 encoding a putative EARLY FLOWERING 4 (ELF4) protein involved in the regulation of plant circadian clock synchronized by environmental cues [29]; Rc07G017352 encoding a member of NIGHT LIGHT-INDUCIBLE AND CLOCK-REGULATED family (LNK2) which plays a role in circadian rhythms, photomorphogenic responses, and photoperiod-dependent flowering time in *Arabidopsis* [30]; and Rc06G012897 encoding PHYTOCHROME B (PHYB) involved light-regulated circadian rhythm and plant growth (Fig. 3f and Additional file 1: Fig. S11) [31]. Additionally, many stress-related genes including disease resistance proteins and heat-shock proteins were identified (Additional file 2: Table S12). These divergent genes between WK and WE may be important for local adaptation and could be used in the future for the diversification of castor bean germplasm.

### A scan for selective sweeps reveals potential targets of selection during domestication

**Fig. 4:**
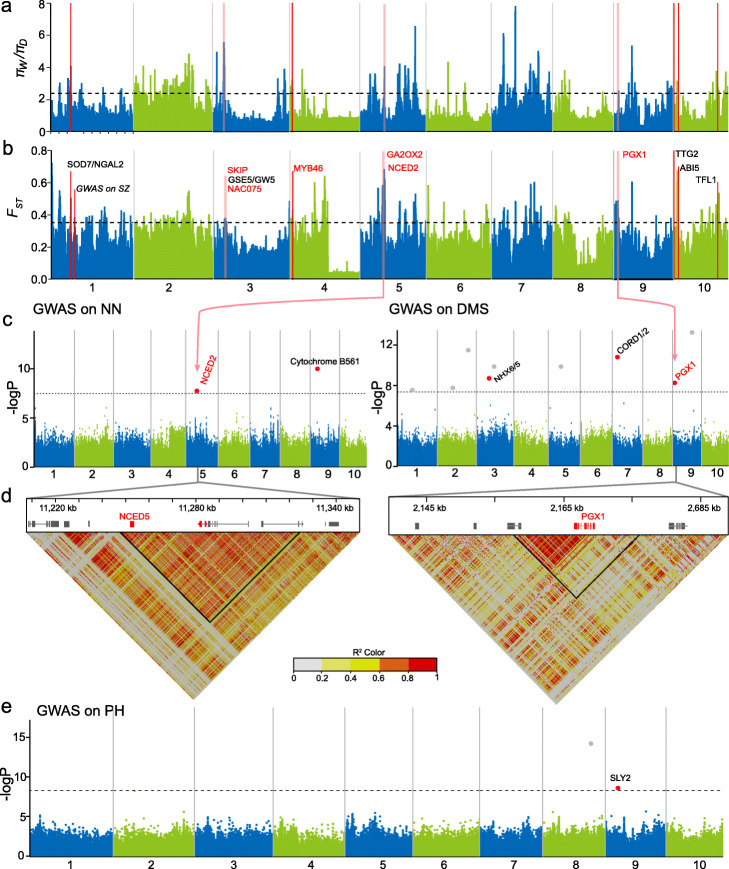
Selective sweep and GWAS analyses on plant architecture in castor bean. **a**, **b** Selective sweep regions identified by **a** the greatest reduction of diversity (ROD) and **b** the greatest relative divergence (*F*_*ST*_) between wild (W) and domesticated (D) populations. Dashed lines indicate the top 5% of the ROD or *F*_*ST*_ values, and red vertical bars indicate known genes involving in the regulation of plant architecture and seed size mentioned in the text. **c** Local Manhattan plots obtained from GWAS for the number of nodes (NN, MLMM model) and diameter of the main stem (DMS, FarmCPU model). Dashed lines indicated the threshold for GWAS (−logP = 7.67). Red lines show two GWAS signals that overlap with selective sweeps in **a** and **b**. Candidate genes closest to the GWAS signals are displayed. **d** Heatmap of LD surrounding the GWAS signals in **c**. Black triangles indicate the positions of the LD block and the color key indicates *r*^*2*^ values between SNPs in the regions. **e** Manhattan plot obtained from GWAS of plant height (PH, MLMM model). Dashed lines indicate the threshold for GWAS (−logP = 7.67). A candidate gene closest to the GWAS signal is displayed

During domestication, several key agronomic traits such as plant height (PH), diameter of the main stem (DMS), number of nodes (NN), and seed size have been selected on by humans, reflecting morphological change from a perennial woody tree to an annual semi-woody crop (Additional file 1: Fig. S12). We employed two metrics, ROD and *F*_*ST*_, to identify potential selective sweeps associated with domestication by comparing the wild population (comprising both WE and WK) with the LC population. This was carried out using a 100-kb sliding window with a 20-kb step. In total, 326 potential selective sweeps in the top 5% of both the ROD and *F*_*ST*_ distributions were detected, making up 4.4% (14.7 Mb) of the assembled genome (Fig. 4a, b). These regions contained 1220 genes (Additional file 2: Table S13) with functions relating to binding, metabolic process, cellular process, biological regulation, localization, and response to stimulus (Additional file 2: Table S14). Many well-studied genes involved in the regulation of flowering, cell wall synthesis, and adaptation were identified, such as Rc10G022330 encoding a putative orthologue of TERMINAL FLOWER 1 (TFL1) that plays a critical role in the regulation of inflorescence meristem identity, flowering time, and plant height in *Arabidopsis* [32, 33]; Rc03G005883 encoding a putative SNW/SKI-interacting protein (SKIP) that involved into the regulation of environmental fitness and floral transition in *Arabidopsis* [34]; Rc03G005826 encoding a NAC transcription factor, homologous to *Arabidopsis* ANAC075 that functions as a repressor of flowering and involved in secondary cell wall formation [35, 36]; Rc04G007260 encoding a member of the R2R3 gene family (MYB46), which involved in the control of secondary cell wall thickening [37]; Rc05G010832 encoding a gibberellin 2-oxidase (GA2OX2) which had a functional role in the control of plant growth and height by regulating GA concentrations in aspen trees [38]; and two genes, Rc02G003752 and Rc02G003753, putatively encoding gibberellin-regulated proteins (Additional file 2: Table S13).

Several genes with orthologues in other species involved in the regulation of seed size were identified (Fig. 4b and Additional file 2: Table S13). For example, Rc01G001375 encodes a putative B3 domain transcription factor, orthologous to *Arabidopsis* NGATHA-like protein (SOD7/NGAL2) that regulates seed size by repressing cell proliferation during seed development [39]. Castor bean gene Rc10G022090 encodes a WRKY family transcription factor orthologous to *Arabidopsis TRANSPARENT TESTA GLABRA2* (*TTG2*) which can regulate endosperm/seed growth by increasing integument cell elongation [40]. The gene Rc03[G]005861 encodes a putative orthologue of rice *GSE5/GW5* which appears to have a crucial function in determining grain width and weight in rice [41]. Finally, Rc10G022347 encodes a bZIP transcription factor, orthologous to *ABSCISIC ACID-INSENSITIVE5* (*ABI5*), which regulates seed size by repressing the expression of *SHORT HYPOCOTYL UNDER BLUE1 (SHB1)* during early seed development [42]. These genes represent strong candidates for follow-up work to determine the genes involved in important aspects of castor bean domestication and agronomically important traits.

### Genome-wide association study (GWAS) reveals the genetic basis of agronomic traits

#### GWAS for plant architecture

Our GWAS identified 13 SNPs significantly associated with three plant architecture traits, including two for NN, nine for DMS, and two for PH (Additional file 1: Fig. S13 and Additional file 2: Table S15). For NN, one SNP association fell within a putative domestication sweep which included the gene GA2OX2 mentioned above and Rc05G010848 encoding a putative 9-cis-epoxycarotenoid dioxygenase (NCED5/3) (Fig. 4c and Additional file 2: Table S16). The gene *NCED5/3* is close to a significant GWAS signal and within a high LD region (Fig. 4c, d) and fine-tunes ABA biosynthesis in *Arabidopsis* [43]. The second SNP significant for NN was located on chromosome 9 in the vicinity of Rc09G020408, an orthologue of *Cytochrome b563* with unknown function*.*

For DMS, nine signals on six chromosomes (1, 2, 3, 5, 7, and 9) were identified (Fig. 4c and Additional file 2: Table S15) and the signal at position 2,179,317 on chromosome 9 overlaps with a domestication sweep (Additional file 2: Table S16). The gene within the domestication sweep that is closest to the GWAS signal is Rc09G019869 which is also in a high LD block (Fig. 4d). This gene encodes an orthologue of POLYGALACTURONASE INVOLVED IN EXPANSION (PGX1), involved in cell walls and cell elongation in *Arabidopsis* [44]. On chromosome 3, the gene closest to a SNP significantly associated with DMS was Rc03G006483, which is orthologous to *Arabidopsis* Na^+^/H^+^ ANTIPORTER 6 and 5 (NHX6/5), and its mutant in *Arabidopsis* is smaller and exhibits slowed development [45]. An additional gene close to a SNP significantly associated with DMS was Rc07G015461 on chromosome 7 orthologous to *Arabidopsis* CORD2 (CORTICAL MICROTUBULE DISORDERING2) which is required for secondary cell wall patterning in xylem vessels [46]. Other candidate genes located nearby the associated signals for DMS were identified, but their putative functions remain unknown due to an absence of annotation (Additional file 2: Table S15).

For PH, we detected two significant SNPs, one each on chromosomes 8 and 9 (Fig. 4e and Additional file 2: Table S15). The SNP on chromosome 9 is located in an intron of Rc09G020376, which putatively encodes the F-box protein SLY2. In *Arabidopsis*, SLY1 and 2 make up the SCF E3 ubiquitin ligase involved in DELLA protein degradation to modulate the GA signaling, and their knockout mutants exhibit a dwarfed phenotype [47, 48].

#### GWAS and QTL of seed size and weight

**Fig. 5:**
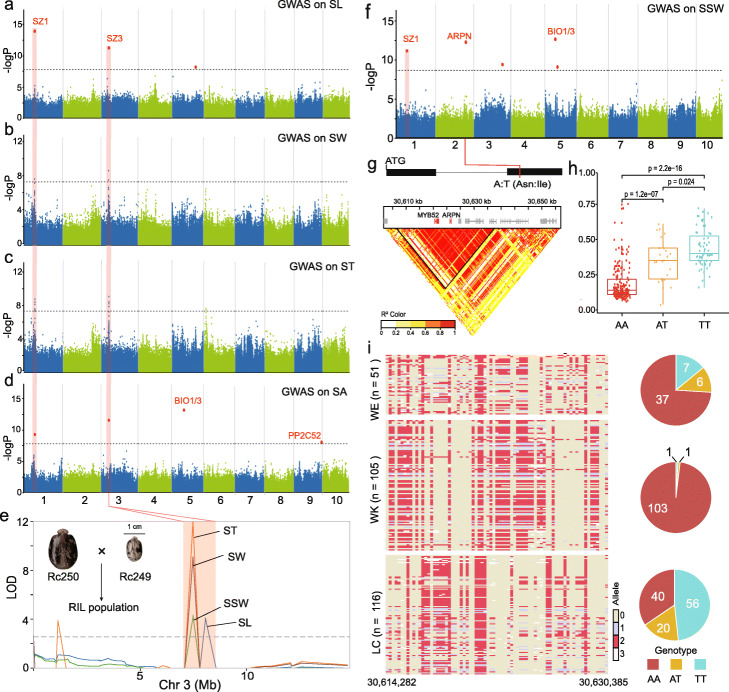
GWAS and identification of candidate genes responsible for seed size in castor bean. **a**–**d** Manhattan plots for GWAS of seed length (SL, MLMM model), seed width (SW, MLM model), seed thickness (ST, MLM model), and seed area (SA, MLMM model). Two shared GWAS signals (SZ1 and SZ3) are shown. The dashed horizontal line indicates the threshold for GWAS (−logP = 7.67). **e** QTL mapping analysis of seed size (including SL, SW, ST, SSW) using recombination inbred lines (RILs) from a cross between landrace Rc250 with large seeds with the cultivar Rc249 with small seeds. The common QTL that overlapped with GWAS signal SZ3 on chromosome 3 is displayed. The dashed horizontal line indicates the LOD threshold for QTLs (LOD = 2.5). **f** Manhattan plots for GWAS of single seed weight (SSW, MLMM model). The dashed horizontal line indicates the threshold for GWAS (−logP = 7.67). The SZ1 signal (see panels **a**–**d**) was identified and two candidate genes are shown. **g** A non-synonymous SNP in ARPN and heatmap of LD. The black triangle indicates the positions of an LD block which contains two genes *MYB52* (Rc02G003980) and *ARPN* (Rc02G003981). The color key indicates *r*^*2*^ values in the LD region. **h** Comparisons of SSW between haplotypes in the GWAS populations. Those carrying the haplotype AA exhibit significantly lighter seeds than those carrying the alternate haplotypes. **i** Allelic distribution surrounding the significant SNP on chromosome 2 in WE, WK, and LC population. The pie charts indicate that the wild populations (WE and WK) contained only a few accessions carrying the haplotypes for large seeds, while the domesticated population (LC) contains many accessions carrying the haplotype for large seeds but some for small seeds

We sought to dissect the molecular basis of seed traits including seed length (SL), width (SW), thickness (ST), area (SA), single seed weight (SSW), and seed oil content (SOC) in castor bean. We first performed QTL analysis using a recombinant inbred line (RIL) population previously constructed by crossing large-seeded line Rc250 with small-seeded line Rc249 [49]. We identified 18 QTLs for five of these six seed traits (none was identified for SA; Additional file 2: Table S17). The GWAS analysis identified 17 GWAS signals associated with five seed traits (none was identified for SOC; Fig. 5 and Additional file 2: Table S15). Notably, there were two genomic regions which were associated with multiple seed traits and we named them as SZ1 (on chromosome 1) and SZ3 (on chromosome 3, Fig. 5). Within SZ1, there were two SNPs (position 11,630,687 and 11,639,673) which were significantly associated with all five traits, while a SNP in the SZ3 region was associated with four traits (Fig. 5a–d, f). SZ1 overlapped with a domestication sweep (Fig. 4b and Additional file 2: Table S16); however, several genes in this region lacked functional annotation or known protein domain (Additional file 2: Table S16). Within SZ1, a significant GWAS signal (position 11,630,687) was located in the intron of Rc01G001604 which encodes a protein of 132 amino acids with unknown function. While genome region SZ3 did not overlap a putative domestication sweep, it does overlap with QTLs for SL, SW, ST, and SSW (Fig. 5e and Additional file 2: Table S18). Previous GWAS on seed length and volume identified the same candidate locus in a Chinese castor bean population [16]. Colocalization of seed size-related trait GWAS signals and QTLs suggest pleiotropy or physical linkage of genes controlling these aspects of seed size in castor bean; however, we also note that some traits are correlated (e.g., SW, ST, and SA; Additional file 1: Fig. S14) and this could be resulting in the co-location of GWAS signals. In the flanking region of SZ3, we identified a microRNA, miRNA396, and gene Rc03G006134. MiRNA396 is implicated in the regulation of seed size and yield in rice [50, 51] and Rc03G006134 encodes an orthologue of the transposase-like DAYSLEEPER gene in *Arabidopsis* and is essential for normal plant growth, especially cotyledon development [52]. The gene Rc05G010958 near the flanking region of the GWAS signal for SA and SSW (Fig. 5d, f) encodes a bifunctional enzyme (BIO1/3) that is involved in biotin synthesis and required for embryo development in *Arabidopsis* [53]. Close to the GWAS signal for SA on chromosome 9, we identified Rc09G021982 encoding a myristoylated 2C-type protein phosphatase (PP2C52), the protein product of which can interact with AGB1 [54], an *Arabidopsis* heterotrimeric G protein β subunit involved in the control of seed size [55]. One member of the protein phosphatase 2C family, PP2C-1 in soybean, has a critical role in the positive regulation of seed size [56].

Seed weight is a critical character for yield, seed germination, and seedling fitness, therefore is a trait that humans likely selected on during domestication. We identified five SNPs significantly associated with SSW on chromosomes 1, 2, 3, and 5 (Fig. 5f and Additional file 2: Table S15). The significant SNP on chromosome 2 is a non-synonymous SNP in the conserved phytocyanin-like domain (PF02298) of a putative phytocyanin protein (ARPN, Rc02G003981), a blue copper protein (Fig. 5). ARPN is the target of miRNA408 that plays an important role in regulating biomass and seed yield in both *Arabidopsis* and rice [57, 58]. ARPN and the gene Rc02G003980, encoding a member of the R2R3-MYB transcription factor family, are in a LD block (Fig. 5g). We found that castor bean accessions carrying the AA haplotype at the SNP in ARPN exhibit significantly lighter seeds than those carrying other haplotypes (Fig. 5h). To further dissect whether these haplotypes for SSW were associated with castor bean domestication, we compared the SNPs within a ~ 16-kb region from 30,614,282 to 30,630,385 bp on chromosome 2 that contains the SNP in ARPN (Fig. 5i). We found that ~ 74% of WE accessions and ~ 98% of WK accessions shared the “small seed” AA haplotype, while there are very few accessions with the “large seed” TT haplotype at SNP2 (7/50 in WE and 1/105 in WK). By contrast, ~ 34% of LC accessions had the “small seed” haplotype, and the remainder had the heterozygous or “large seed” haplotypes (Fig. 5i). In sum, these results provide important information to progress our understanding of the genetic basis of architectural and yield-association traits, with significant implications for future breeding of castor bean.

## Discussion

Most investigations of crop domestication have focused on food crops; little is been known about the cultivation, domestication, and genetic variation in domesticated non-food crops, with the exception of cotton [59]. Castor bean has been used in human society for at least several thousand years due to the unique fatty acid oils (for lighting) [60] and the toxic protein ricin (for hunting) [7] which accumulate in its seeds. To extend our knowledge of the domestication of non-food crops, we have assembled a chromosome-scale genome of wild castor bean, examined genomic variation, and started to dissect the genetic basis of key architectural traits during the domestication of castor bean from a woody tree to an annual oilseed crop.

**Fig. 6:**
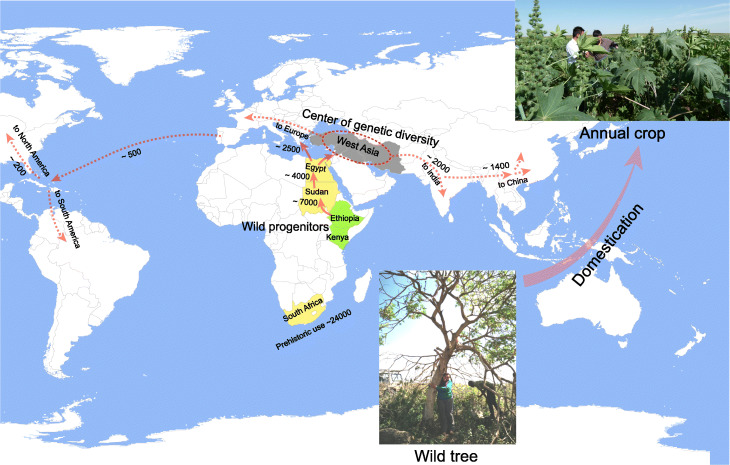
Proposed dispersal of castor bean worldwide reconstructed from population genomics, and archeological records. Inset photographs show the domestication process from a woody tree originally distributed in East Africa (pale green) to an annual crop grown throughout the world. Accessions from East Africa are the extant wild progenitors of castor bean. Archeological evidence suggests prehistoric use of castor bean in South Africa and the early managements in Sudan and Egypt which subsequently spread to Europe. Western Asia (including Turkey, Syria, Iraq, and Iran; gray area) represents the *center of genetic diversity of landraces and cultivars*, from which cultivated castor bean likely spread to India and China. The movement of castor bean from Europe to America was a recent event

Using our newly assembled wild castor bean genome, we uncovered details of genome evolution in the Euphorbiaceae, identifying chromosome fusions, fissions, translocations, and a whole-genome duplication. By resequencing and analyzing 505 accessions collected worldwide, covering wild, landrace, and cultivated castor bean, we reveal that accessions from East Africa are the extant wild progenitors of castor bean. Furthermore, we found that there is genetic differentiation between wild castor bean from Kenya and Ethiopia, with this divergence estimated to have occurred ~ 7000 YBP, coincident with dramatic climate change associated with the Turkana depression in the East African rift during the Mid-Holocene [26]. This dramatic change in climate coincides with the genetic bottleneck in wild castor bean populations as evidenced by a sharp reduction of effective population size around 4000–6000 YBP. We found that the domestication of castor bean occurred ~ 3200 YBP, and West Asian landraces and cultivars exhibited high genetic diversity. From this, we infer that domestication occurred somewhere between our East African collection sites and West Asia; however, an absence of wild material outside East Africa and an absence of landrace and cultivar accessions from Northern Africa (especially Egypt and Sudan) mean that we lack a more precise location of domestication (Fig. 6). Since then, the domesticated castor bean was introduced initially to Europe (~ 2500 YBP) and India (~ 2000 YBP), and spread to China later (~ 1400 YBP) [9], consistent with the decline of genetic diversity from West to East Asia. The introduction to America of castor bean may have occurred after the discovery of the new continent by Columbus (~ 500 YBP) [9]. Accessions from Sudan and Egypt are poorly represented in seed banks yet are likely to represent transitional forms crucial to understanding castor bean domestication and the location of early management or domestication and should be considered a future target for seed collections.

During castor bean domestication, a significant evolution of plant architecture is evidenced from a perennial tall woody tree to an annual and dwarfed crop (Fig. 6). Combining GWAS and QTL analyses allowed us to investigate the genetic basis of a range of traits, especially those related to plant architecture and seed size, another clear target of selection. We detected diverse genes which were likely targets of selection with putative functions related to the regulation of stem growth, flowering, secondary cell wall synthesis, and links to genes involved in seed development from other species. We find genetic structure within the wild germplasm and this is at least in part related to ecology; therefore, this may endow wild castor bean with considerable adaptive variation ripe for the future breeding of adaptable castor bean varieties. Overall, this study not only generates new insights into the origin of castor bean, and its dramatic morphological evolution from a wild perennial woody tree to a cultivated annual crop, but also serves as a resource for the genetic improvement of this important crop.

## Conclusions

We provide evidence of adaptive population divergence in wild castor bean and identify demographic and genomic patterns associated with the transition into dwarfed annual castor bean crop, a unique and important non-food plant used by human in prehistory and today. Sequencing and assembly of the genome of a wild progenitor and resequencing of wild populations, cultivars, and landraces revealed that East African castor bean represents the extant wild progenitors, and domestication of castor bean occurred ~ 3200 years ago. By identifying candidate genes associated with plant architecture and seed traits, our study provides novel insights into the understanding of domestication and genome evolution of castor bean, with implications for other non-food and tree crops.

## Methods

### Sample collection and plant materials

In this study, we utilized seeds of 280 castor bean accessions from 35 countries, covering the worldwide distribution of castor bean. This comprised seeds of 222 castor bean accessions we collected, including 155 wild accessions from Ethiopia and Kenya, with the assistance of the World Agroforestry Center (ICRAF), and 67 landraces and cultivars from Pakistan, India, and China. The remaining 58 accessions were kindly provided by USDA-Agricultural Research Center in Griffin, Georgia. Detailed information for each accession is listed in the Additional file 2: Table S7.

The seed was sterilized and germinated in an incubator at 30 °C for 5 days. Subsequently, germinated seeds were transplanted into our experimental field in Kunming, Yunnan, China. Five individuals were planted for each accession. For phenotypic observation and subsequent GWAS, we attempted to grow all accessions in the same field in two consecutive years from 2017 to 2018. In the first year, all castor bean accessions were self-pollinated. Accessions with consistent phenotypes across individuals (mainly referring to the stem color and seed size) were then cultivated in the second year; however, ~ 35% were lost due to uncertain climatic factors. Hence, some accessions’ data is based on 1 year of cultivation, but for most, it is averaged across 2 years. Young leaf tissue was collected in the second year from one individual of each accession for subsequent genome resequencing. In addition, we downloaded WGS data of 225 castor bean lines with clear collection information from the NCBI database under SRA accession number PRJNA548999 (Additional file 2: Table S7) [16]. In total, 505 castor bean accessions were used for subsequent analyses.

### Genome sequencing and assembly

We selected a wild accession “Rc039” from Anabora District, Ethiopia (8° 3′ N, 38° 9′ E), that displays traits typical of wild castor bean. Genomic DNA was extracted using the Plant Genomic DNA Kit (TIANGEN, Beijing, China), and libraries were constructed and sequenced on the NovaSeq 6000 platform. The raw reads were pre-processed to remove the adaptors and low-quality bases using fastp (version 0.20.0) [61] with parameter min-length 75. K-mer distribution was estimated using jellyfish (version 2.2.6) [62] with parameters “-m 17 -C,” and genome size was estimated with GenomeScope [63]. Genome size was also estimated by flow cytometry using maize B73 (~ 2300 Mb) as an internal standard.

For de novo assembly of the Rc039 genome, we used long read sequencing on the PacBio Sequel platform with two SMRT Cells. In brief, high molecular weight (HMW) DNA was used to construct a DNA library with ~ 20 kb insert size and subsequently sequenced on the PacBio Sequel sequencing platform at Shanghai OE Biotech Co., Ltd. (Shanghai, China). De novo assembly was performed with FALCON (pb-assembly version 0.3.0) [64] with the following parameters: *length_cutoff = − 1*, *seed_coverage = 40*, *length_cutoff_pr = 12 Kb*, *pa_HPCdaligner_option = -v -B128 -M20 -T8*, *pa_daligner_option = -e0.75 -l4800 -k18 -h480 -w8 -s100*, *ovlp_daligner_option = -k24 -h1024 -e.96 -l2400 -s100*, *ovlp_HPCdaligner_option = -v -B128 -M24 -T8*, *falcon_sense_option = --output_multi --min_idt 0.70 --min_cov 3 --max_n_read 300*. Subsequently, the contigs were phased and polished by FALCON-Unzip based on all PacBio long reads. Finally, the assembled contigs were filtered to remove potential contaminants by BLASTN against NCBI NT database with the parameters *-evalue 1e-5 -best_hit_overhang 0.25 -perc_identity 0.5 -max_target_seqs 10*. Finally, sequence polishing was performed with Arrow (https://github.com/PacificBiosciences/GenomicConsensus, version: 2.3.3) using PacBio long reads, and then Pilon (version: 1.23) [65] using Illumina short reads.

### Hi-C sequencing and gap filling

The Hi-C sequencing library was constructed and sequenced (150-bp paired-end) on the NovaSeq 6000 platform. Raw reads were quality-trimmed with fastp as mentioned above and aligned to the draft genome assembly using Juicer [66] with default parameters and a chromosome-scale assembly was generated using 3D de novo assembly (3D-DNA) pipeline [67] (https://github.com/theaidenlab/3d-dna) with the parameters -*r 1 -q 10*. The resulting assembly was visualized using Juicebox Assembly Tools (version 1.11.9) [68] based on a contact matrix, and the mis-assemblies and mis-joins were manually corrected based on neighboring interactions. After scaffolding, we employed PBJelly in the PBSuite package (version 15.8.24) [69] to close gaps between contigs. Finally, we performed the second-round error correction as mentioned above. The completeness and accuracy of genome assembly were quantitatively assessed by BUSCO (version 3.1.0) [70] with the eudicot odb10.

### Genome annotation

For repeat annotation, we adopted the Extensive *de-novo* TE Annotator (EDTA version 1.7.0) [71], which incorporates LTRharvest, LTR_FINDER, LTR_retriever, TIR-Learner, HelitronScanner, RepeatModeler, and RepeatMasker, as well as customized filtering scripts for de novo identification of each TE class, and compiles the results into a comprehensive TE library. Subsequently, the TEs identified were annotated by searching the EDTA TE library using RepeatMasker (version 4.0.9) [72].

Protein-coding annotations were predicted using the MAKER (version 2.31.10) [73] annotation pipeline which integrated ab initio prediction, RNA-seq, and homology-based approach based on the masked genome. For ab initio prediction, we used the gene predictor software Augustus (vers. 3.3.2) [74] and GeneMark-ES (version 4.3.8) [75] which were previously trained using BRAKER2 [76] (https://github.com/gatech-genemark/BRAKER2) with RNA-Seq data (four samples including root, stem, leaf, and seed, ~ 6Gb clean reads for each sample). These samples were also aligned to the genome using HISAT2 (version 2.10.2) [77] and transcripts were reconstructed by StringTie (version 1.3.0) [78]. The transcripts from the RNA-seq, 62,629 expressed sequence tags (castor bean EST, download date: 2019-04-17, NCBI), and protein sequences from six plant species: *Hevea brasiliensis*, *Manihot esculenta*, *Ricinus communis* “Hale”, *Arabidopsis thaliana* (all downloaded from phytozome12: https://phytozome.jgi.doe.gov/pz/portal.html), *Vernicia fordii* (downloaded from http://bigd.big.ac.cn/gsa, GWHAAEU00000000), and *Jatropha curcas* (downloaded from China National GeneBank under accession number CNP0000449) were used as evidence during annotation, and finally to generate a comprehensive set of protein-coding genes with a AED score [19]. BUSCO [70] was used for the evaluation of annotation completeness with eudicotyledons_odb10. Approximately 96.0% of conserved genes (2036/2121) were identified in the castor bean genome. In addition, we also predicted noncoding RNAs (rRNA, small nuclear RNA, and microRNAs) using RNAmmer (version 1.2) [79] and Infernal (version 1.1.2) [80] by searching Rfam (version 14.1) [81]. The tRNAs were identified using tRNAscan-SE (version 1.3.1) [82].

Functional annotations were assigned by aligning the castor bean protein sequences to the public databases including SwissProt, TrEMBL, NR, eggNOG, and KOG databases using diamond (*E*-value ≤1e^−5^). Motifs and domains were annotated by searching ProDom, PRINTS, Pfam, SMRT, PANTHER, and PROSITE using InterProScan (version 5.36). Gene Ontology (GO) annotations were assigned according to the corresponding InterPro entry.

### Comparative genome analyses

Protein sequences from ten eudicot genomes: *Hevea brasiliensis*, *Manihot esculenta*, *Populus trichocarpa*, *Linum usitatissimum*, *Arabidopsis thaliana*, *Glycine max*, and *Vitis vinifera* (downloaded from phytozome12: https://phytozome.jgi.doe.gov/pz/portal.html), *Vernicia fordii* (downloaded from http://bigd.big.ac.cn/gsa), *Mercurialis annua* (downloaded from https://osf.io/a9wjb/), and *Jatropha curcas* (downloaded from China National GeneBank under accession number CNP0000449) were obtained. Orthologous genes among these plant species and castor bean were identified using OrthoFinder2 (version 2.2.7) [83] with the parameter *-S diamond*. Subsequently, all single copy orthologs were subjecting to multiple sequence alignment using MAFFT (version 7.407) [84] and poorly conserved blocks were trimmed using trimAl [85] with default parameters. Finally, the consensus sequence was merged into a supergene. The phylogenetic tree was constructed using RAxML (version 8.1.2) [86] with 100 bootstrap replicates and PROTGAMMAAUTO model. Divergence times were estimated under a relaxed clock model using MCMCtree in PAML (version 4.9i) [87] with the following parameters: *burnin = 1,000,000*, *nsample = 20,000*, and *sampfreq = 500*, and divergence dates for *Vitis vinifera* (105–115 MYA), *Glycine max* (97–109 MYA), and *Arabidopsis* (75–99 MYA) obtained from Timetree (http://www.timetree.org/) were further used to calibrate the divergence time. Evolutionary analysis of gene synteny and collinearity were performed using MCScan (python version, https://github.com/tanghaibao/jcvi/), and syntenic gene pairs were visualized using the dotplot script in jcvi package. We used CAFE (version 4.2) [88] to identify the expansion and contraction of gene family in castor bean genome relative to other plant species. Whole-genome duplication (WGD) was detected by corrected fourfold synonymous third codon transversion (4DTv) with an in-house perl script and synonymous substitution rate (Ks) calculated with the NG model in KaKs_Calcuator (version 2.0) [89].

### Genomic resequencing and variant calling

Genome resequencing was carried out for 280 castor bean accessions using the same methods as above for the Illumina NovaSeq 6000 samples. Combined with the WGS data mentioned above [16], the clean reads from 505 accessions were mapped to the Rc039 genome using bwa-mem (version 0.7–17) [90] with default parameters. Picard tools (version 2.18.17, http://broadinstitute.github.io/picard/) were used to remove PCR duplicates according to the mapping coordinates. Genetic variants including SNPs and Indels (short insertion and deletion) were detected using Genome Analysis Toolkit (GATK, version 3.8.1) [91] and its subcomponents HaplotypeCaller, CombineGVCFs, and GenotypeGVCFs to form a merged vcf file with all samples. SNPs were filtered with the following parameters: *QD < 2.0*, *MQ < 40.0*, *FS > 60.0*, *SOR > 3.0*, *MQRankSum < − 12.5*, *ReadPosRankSum < − 8.0*, and indels filtered with the parameters *QD < 2.0*, *FS > 200.0*, *MQ < 40.0*, *SOR > 10.0*, *ReadPosRankSum < − 20.0*. From this, we defined a core SNP set by removing SNPs with more than two alleles, > 20% missing calls and MAF < 1% which was used for subsequent analyses.

According to the gene model of the Rc039 genome, genetic variants identified above (SNPs and indels) were further annotated using the SNPeff (version 4.3T) [92], and the density across each chromosome was determined with 500-kb sliding windows using VCFtools (version 0.1.17) [93].

### Population genetic diversity and structure analysis

To infer the basal group of castor bean, we constructed a rooted phylogenetic tree based on 48,450 SNPs from 9063 single copy orthologs between castor bean and *Jatropha curcus*. Briefly, all single copy orthologs between castor bean Rc039 and *J. curcas* were identified using the OrthoFinder2 [83] with default parameters, and single copy orthologs were obtained for each castor bean accession by replacing the corresponding SNPs. The resulting single copy orthologs were then merged into a supergene and a rooted maximum likelihood tree was constructed using IQ-TREE (version 1.6.12) [94] with the parameters -alrt 1000 -bb 1000 GTR+F+R2 (ultrafast bootstrap) with *J. curcus* as outgroup. The phylogenetic tree was visualized using the R package ggtree [95].

Based on the phylogenetic analyses, we defined three groups of individuals: WE (wild accessions from Ethiopia), WK (wild accessions from Kenya), and LC (landrace and cultivated accessions from throughout the world). Nucleotide diversity (*θ*_*π*_) was determined for WE, WK, and LC population using VCFtools [93] using a 100-kb sliding window with a 20-kb step size. Genetic differentiation (*F*_*ST*_) was calculated among different groups using the same method. To detect selective sweeps, we calculated the ROD and *F*_*ST*_ value (wild vs. cultivar) within the same sliding windows and the regions that scored in the top 5% of the ROD and *F*_*ST*_ values were defined as candidate domestication sweeps. LD decay for each population was estimated for all pairs of SNPs using PopLDdecay (version 3.4) [96] with the parameters *−MAF 0.05 −Het 0.88 −Miss 0.25 −MaxDist 300*.

Before inferring the population structure, we pre-processed the core SNP set by adopting a linkage disequilibrium pruning procedure using PLINK [97] with parameters *indep-pairwise 50 10 0.5.* In total, we obtained 754,561 SNPs that were used for subsequent analyses. Population structure was performed using ADMIXTURE (version 1.3) [98] with a block-relaxation algorithm with the core SNP set, and the genetic ancestry of each sample was estimated by specifying the number of genetic clusters (*K*) from 2 to 20 and running the cross-validation error (CV) procedure (Additional file 1: Fig. S7). We carried out PCA using EIGENSOFT (version 6.1.4) [99] with default parameters and the first two eigenvectors were plotted.

In order to further understand population splits and mixtures in castor bean, we employed TreeMix [100] to construct a subgroup graph based on the core SNP set. TreeMix runs were conducted 8 times allowing for 0–8 admixture events (m). The model with the optimal number of admixture events, m = 6, was chosen based on the explained variance more than 99%, beyond which the explained variance improved only marginally. Bootstrap support for the resulting tree topologies was obtained using 100 bootstrap replicates with PHYLIP [101]. Meanwhile, gene-flow information and migration events were mapped onto this tree.

### Population demographic analysis

We first estimated the mutation rate per nucleotide per year (*μ*) for castor bean. Briefly, we identified syntenic regions between  castor bean and *J. curcas* genomes using LASTZ (version 1.04.03) [102] with the parameters *T = 2*, *C = 2*, *H = 2000*, *Y = 3400*, *L = 6000*, and *K = 2200*. The number of base pair mismatch within syntenic regions was calculated that excluded those with ambiguous nucleotide and within gap region, resulting in the 34.0% sequence divergence between them. We assumed a generation time of 2 years for wild castor bean as observed in our field investigations and a divergence time of 48.8 MYA between castor bean and *J. curcas* (as estimated in the species tree above), giving *μ* = 6.9 × 10^−9^ mutations per nucleotide per year for castor bean, consistent with a previous average estimate for plant nuclear genes ranging from 5 × 10^−9^ to 7 × 10^−9^.

We employed SMC++ (version v1.15) [103] to infer population size histories and split times between two populations based on the unphased SNPs with MAF > 0.05. We performed the masking step as suggested [104] to delineate the largely uncalled regions with SNPable toolkit (http://lh3lh3.users.sourceforge.net/snpable.shtml). The above substitution rate and a generation time of 2 years for wild castor bean or 1 year for cultivated castor bean were used to convert the scaled times and population sizes into real times and sizes, respectively.

We employed environmental niche modeling (ENM) to study the past demographic processes and potential distribution of castor bean from the Last Glacial Maximum (LGM, 21–18 thousand years ago, KYA) to Mid-Holocene (7–5 KYA) and the present. The occurrence sites of castor bean were collected from our field investigations, records, and collection databases (http://www.ars-grin.gov/) and were manually checked to exclude duplicated and illogical sites and cultivated sample sites. We downloaded 19 climatic variables across the three periods mentioned above from the WORLDCLIM database (www.worldclim.org). We further removed four occurrence records that lacked environmental variable data. To reduce the overfitting of these bioclimatic variables on models, environmental variables with Pearson’s correlation coefficient *r* > 0.7 or < − 0.7 were excluded. As a result, eight environmental variables were used for subsequent analysis: Bio1 (annual mean temperature), Bio2 (mean diurnal range), Bio3 (isothermality), Bio5 (max temperature of the warmest month), Bio8 (mean temperature of the wettest quarter), Bio16 (precipitation of the wettest quarter), Bio17 (precipitation of the driest quarter), and Bio19 (precipitation of the coldest quarter). Ecological niche models were performed based on the present variables using the maximum entropy in Maxent (version 3.3.3) [105] with 10 subsample replicated runs and 30 random test percentage.

### Phenotyping and GWAS analysis

Nine agronomic traits were measured in 2017 and 2018 in our experimental field, focusing on those traits that differed between wild and domesticated castor bean. We combined the data from five plants in each of the 2 years and the mean value was used for GWAS analysis. As mentioned above, some accessions did not survive in the second year and hence 1 year of data was used. Because of the 2-year generation time for wild castor bean, we averaged the seed phenotypes of that collected from the maternal plant in the wild as well as the seed phenotypes after one season which were highly consistent. For plant architecture, we measured three traits including plant height (PH) aboveground, diameter of the main stem (DMS), and the number of nodes (NN). Seed traits, including seed length (SL), width (SW), and thickness (ST), were determined by a digital caliper. For seed area (SA), five seeds were first scanned by a scanner and the area was calculated using Adobe Photoshop software. Single seed weight (SSW) was determined as the average value of 30 seeds. The seed oil content (SOC) was measured by MQ-ONE Seed Analyzer (BRUKER, Germany) using NMR. For each phenotypic trait, more than five biological replicates were used in this study.

In total, 2,314,859 SNPs with MAF > 0.05 and present in the 279 phenotyped individuals we cultivated were used for GWAS. GWAS was performed using the MLM, MLMM, and FarmCPU statistical methods implemented in GAPIT (version 3.0) [106]. The first three PCA values (eigenvectors) and kinship (K) matrix generated with GAPIT were used to correct for population structure and random polygenic effect. We identified significant GWAS signals after applying an adjusted Bonferroni test threshold of 7.67, corresponding to a raw *P* value of 2.15 × 10^−8^ based on a nominal level of *α* = 0.05. The LD blocks around GWAS signals were further evaluated by calculating *r*^*2*^ between SNPs using PLINK and visualized using the R package LDheatmap (version 0.99-7) [107].

### Genetic map construction and QTL analysis

We reconstructed a genetic map based on recombinant inbred lines (RILs) by crossing the landrace Rc250 with large seed with the cultivar Rc249 with small seed. The GBS sequencing data from the two parents and 200 offspring were obtained from our previous study [49]. In total, 23,413 high-quality bi-allelic SNPs were called using GATK with the following criteria: (i) QD < 2.0, MQ < 40.0, MQRankSum < − 12.5, ReadPosRankSum < − 8.0; (ii) progeny depth > 8 and GQ > 30; and (iii) missing data in progenies less than 10% and MAF > 0.05. Subsequently, the genetic map was constructed using Lep-MAP3 (version 0.2) [108] and linkage groups (LGs) were defined based on a LOD (logarithm of odds) score of 41 and a fixed recombination fraction of 0.03. We resolved 10 LGs and each LG contained at least 1167 SNPs. The order of markers and the genetic distance were then estimated using Lep-MAP 3 [108] with the parameters *useKosambi = 1 sexAveraged = 1 grandparentPhase = 1*. The final genetic map included 18,946 SNP markers and the total genetic length was 1244.54 cM. This genetic map was used to recalibrate and evaluate the assembly of the Rc039 genome using ALLMAPS [109] with default parameters. In addition, QTL analysis was performed for five seed traits (SL, SW, ST, SOC, and SSW) using the QTL IcIMapping (version 4.2) [110] with 2186 bin markers and significantly associated QTL loci were identified based on a LOD threshold of 2.5.

## Supplementary Information

**Additional file 1: Fig. S1.** Photos of wild castor bean tree from East Africa. **Fig. S2.** Genome size estimate for wild castor bean Rc039. **Fig. S3.** Genome-assisted assembly and chromosome anchoring. **Fig. S4.** The cumulative fraction of Annotation Edit Distance (AED) scores for the assembly of the wild castor bean genome. **Fig. S5.** Distribution of Ks values between syntenic gene pairs among six eudicot species,including *Hevea brasiliensis* (Hbr), *Jatropha curcas* (Jcu), *Manihot esculenta* (Mes), *Ricinus communis* (Rco), *Vernicia fordii* (Vfor) and *Vitis vinifera* (Vvi). **Fig. S6.** A rooted phylogenetic tree of 505 worldwide castor bean accessions based on maximum likelihood with *Jatropha curcas* as outgroup. **Fig. S7.** Population structure analysis in castor bean. **Fig. S8.** Nucleotide diversity in castor bean populations or subgroups. **Fig. S9.** Inferred population splits and admixture of castor bean using TreeMix. **Fig. S10.** Effective population size was inferred by SMC++ based on WGS SNPs data for WE, WK and LC. **Fig. S11.** Two regions of the genome containing the top 5% of *F*_*ST*_ values between WK (wild Kenya) and WE (wild Ethiopia) group. **Fig. S12.** Histogram and boxplot of nine agricultural traits. **Fig. S13.** Quantile-quantile plots for nine agricultural traits by comparing the observed –log_10_P with expected –log_10_P of GWAS. **Fig. S14.** Correlation of five seed traits, seed length (SL), width (SW), thickness (ST), area (SA), single seed weight (SSW). The number and color in the grid indicate the Pearson’s correlation coefficient.

**Additional file 2: Table S1.** BUSCO (Benchmarking Universal Single-Copy Orthologs) evaluation of genome completeness of wild castor bean. **Table S2.** Classification and annotation of repetitive sequences in the wild castor bean genome. **Table S3.** The number and distribution per chromosome of protein-coding genes and non-coding RNAs in the wild castor bean genome. **Table S4.** Functional annotation of wild castor bean genome. **Table S5.** Genes specific to castor bean relative to other five eudicot species (*Hevea brasiliensis*, *Jatropha curcas*, *Manihot esculenta*, *Mercurialis annua* and *Vernicia fordii*). **Table S6.** Kyoto Encyclopedia of Genes and Genomes (KEGG) enrichment for genes that have undergone expansion in castor bean genome. **Table S7.** Detailed information of 505 castor bean lines used in this study. **Table S8.** Information of genome resequencing data and mapping. **Table S9.** Summary of single nucleotide polymorphism (SNP) and insertions and deletions (indels) among 505 castor bean accessions. **Table S10.** Estimation of the relative contributions of the eight environmental variables to the Maxent model for all populations. **Table S11.** Genes identified as potential divergence between WK and WE based *F*_*ST*_ analysis. **Table S12.** Gene Ontology (GO) analysis of divergent genes between WK and WE population. **Table S13.** Domestication-related genes identified by two methods ROD and *F*_*ST*_ analyses. **Table S14.** Gene Ontology (GO) analysis of domestication-related genes. **Table S15.** Genome-wide associated for eight yield-associated traits in castor bean (for SOC no significant SNPs were identified). **Table S16.** Candidate genes within the domestication sweeps overlapping with the GWAS signals. **Table S17.** QTL analysis for five seed traits including seed length (SL), seed width (SW), seed thickness (ST), seed oil content (SOC) and single seed weight (SSW). **Table S18.** Candidate genes within QTL loci (Chr3: 7210428–8,076,805) associated with seed size.

**Additional file 3.** Review history.

## Data Availability

All the sequencing and the genome assembly data in this study have been deposited in NCBI under the BioProject accessions PRJNA706790 [111], which are also available at http://oilplants.iflora.cn. Previously published whole-genome sequencing data for 228 castor bean accessions were downloaded from the NCBI database under the accession number PRJNA563965 [112] and PRJNA548999 [113].
