# Supplementary material for: Genomic insights into the origin, domestication and genetic basis of agronomic traits of castor bean
Source: Genome Biol. 2021 Apr 20;22:113. doi: 10.1186/s13059-021-02333-y (PMC8056531; doi:10.1186/s13059-021-02333-y)
Supplement: Supplementary file 1 — Additional file 1: Fig. S1. Photos of wild castor bean tree from East Africa. Fig. S2. Genome size estimate for wild castor bean Rc039. Fig. S3. Genome-assisted assembly and chromosome anchoring. Fig. S4. The cumulative fraction of Annotation Edit Distance (AED) scores for the assembly of the wild castor bean genome. Fig. S5. Distribution of Ks values between syntenic gene pairs among six eudicot species,including Hevea brasiliensis (Hbr), Jatropha curcas (Jcu), Manihot esculenta (Mes), Ricinus communis (Rco), Vernicia fordii (Vfor) and Vitis vinifera (Vvi). Fig. S6. A rooted phylogenetic tree of 505 worldwide castor bean accessions based on maximum likelihood with Jatropha curcas as outgroup. Fig. S7. Population structure analysis in castor bean. Fig. S8. Nucleotide diversity in castor bean populations or subgroups. Fig. S9. Inferred population splits and admixture of castor bean using TreeMix. Fig. S10. Effective population size was inferred by SMC++ based on WGS SNPs data for WE, WK and LC. Fig. S11. Two regions of the genome containing the top 5% of FST values between WK (wild Kenya) and WE (wild Ethiopia) group. Fig. S12. Histogram and boxplot of nine agricultural traits. Fig. S13. Quantile-quantile plots for nine agricultural traits by comparing the observed –log10P with expected –log10P of GWAS. Fig. S14. Correlation of five seed traits, seed length (SL), width (SW), thickness (ST), area (SA), single seed weight (SSW). The number and color in the grid indicate the Pearson’s correlation coefficient. [file 13059_2021_2333_MOESM1_ESM.docx]

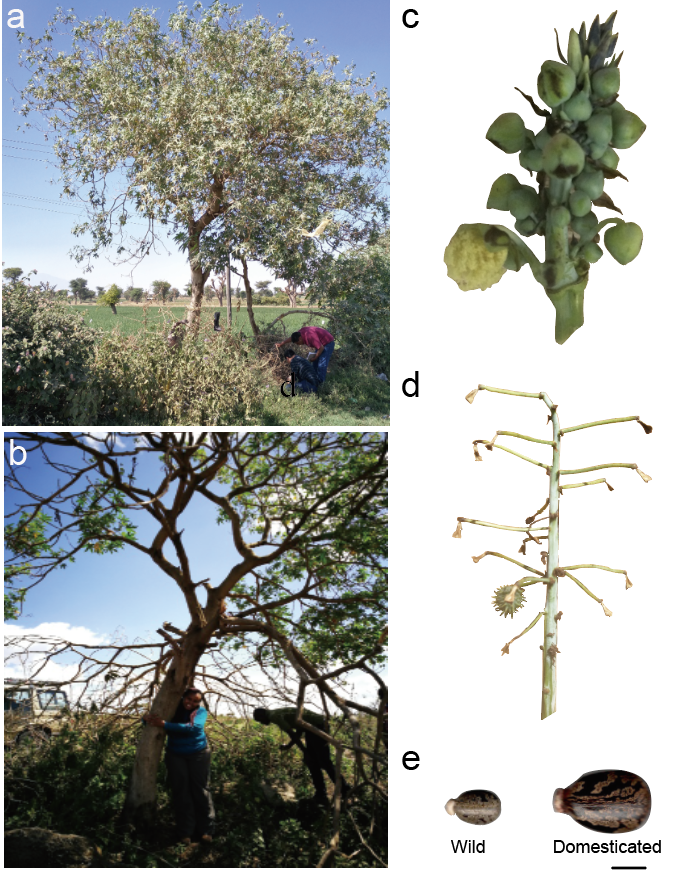
**Figure S1. Photos of wild castor bean tree from East Africa.** (a) Castor bean tree (accession Rc039, reference genome) in Ethiopia. (b) Castor bean (accession Rc102) in Kenya. (c) Young inflorescence of wild castor bean (accession Rc039). (d) Mature inflorescence with shattering fruit in wild castor bean (accession Rc039). (e) Comparison of typical seed size of wild and domesticated castor bean. Scale bar represents 1 cm.


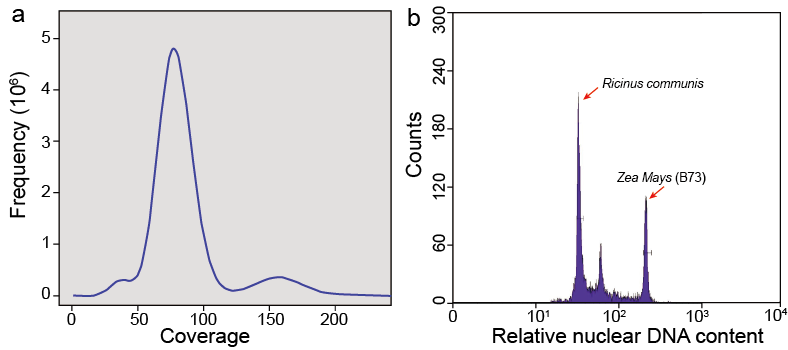


**Figure S2. Genome size estimate for wild castor bean Rc039**. (a) Assessment of genome size and complexity based on *K-*mer method. (b) Estimate of genome size by flow cytometry using *Zea mays* genome (B73; 2,300 Mb) as an internal standard.


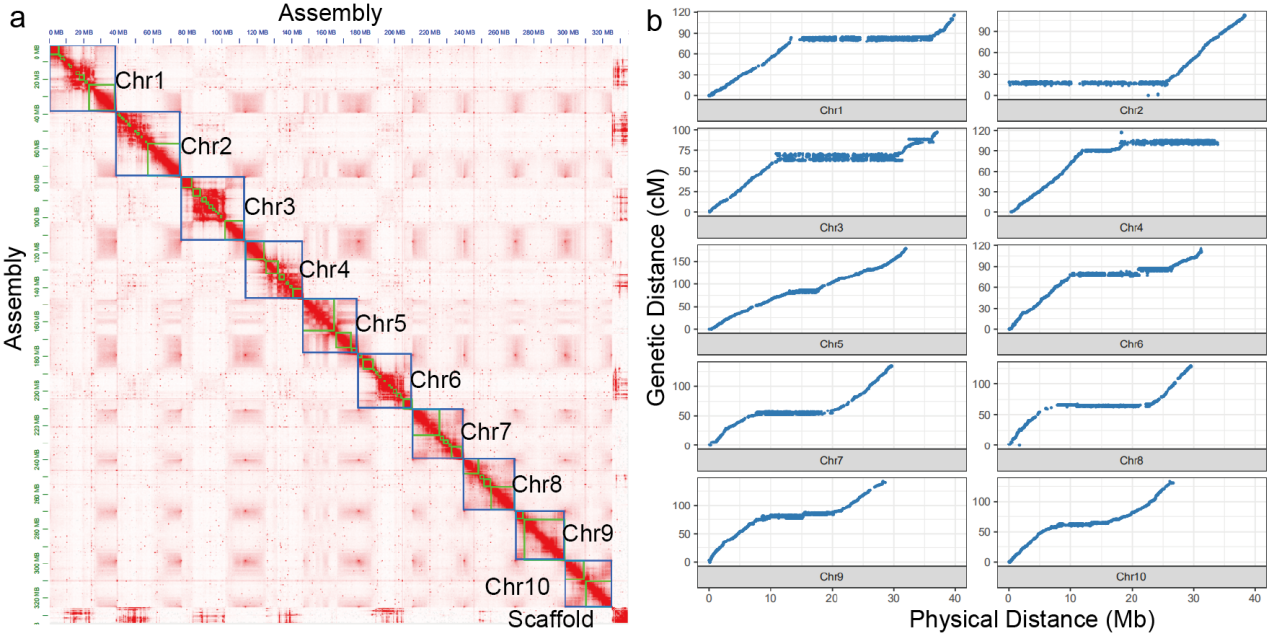


**Figure S3**. **Genome-assisted assembly and chromosome anchoring**. (a) Hi-C map of the castor bean genome. (b) Genetic distance vs. physical distance of each chromosome.


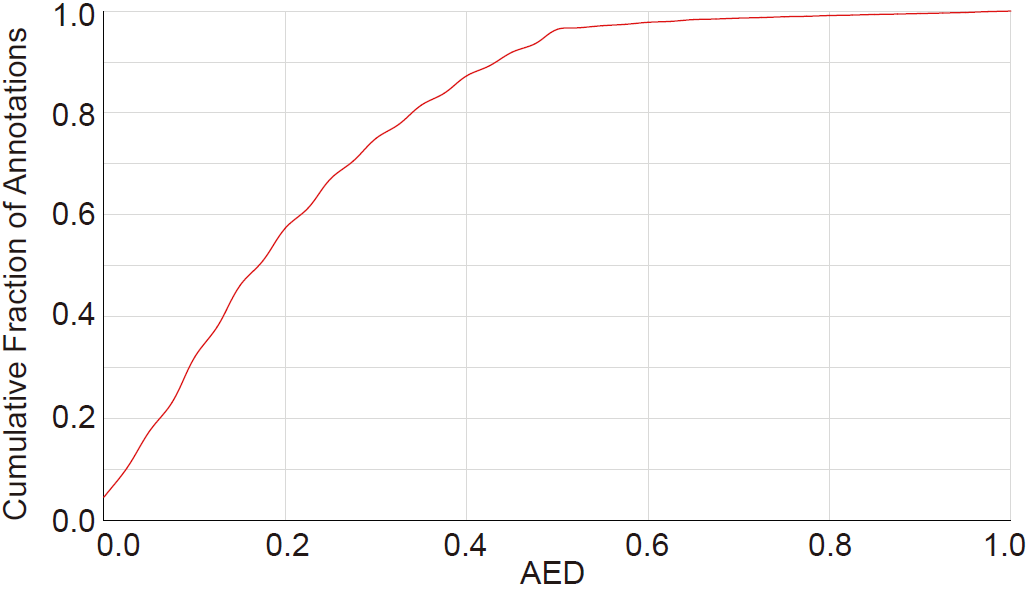


**Figure S4. The cumulative fraction of Annotation Edit Distance (AED) scores for the assembly of the wild castor bean genome.**


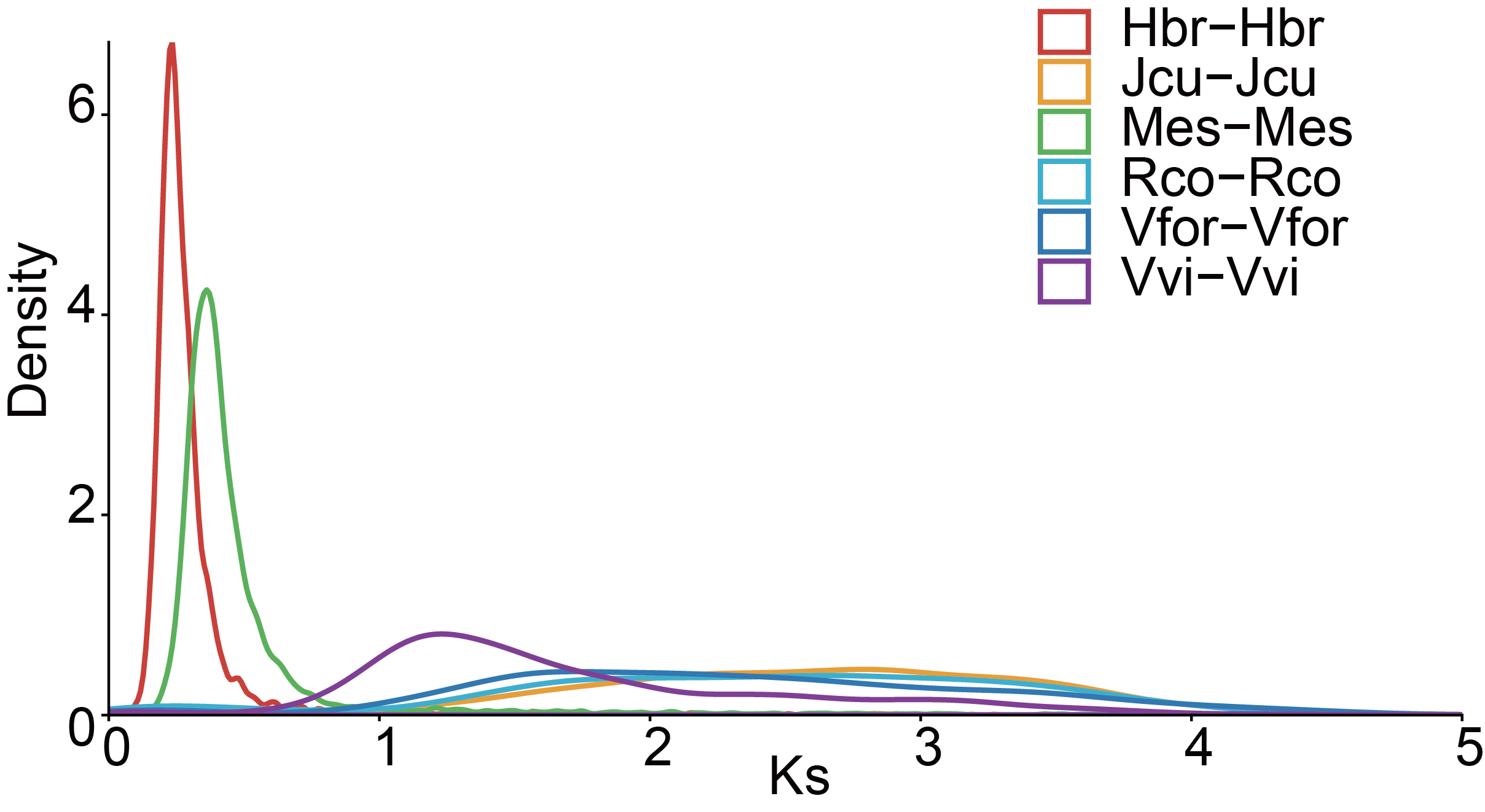


**Figure S5. Distribution of Ks values between syntenic gene pairs among six eudicot species**, including *Hevea brasiliensis* (Hbr), *Jatropha curcas* (Jcu), *Manihot esculenta* (Mes), *Ricinus communis* (Rco), *Vernicia fordii* (Vfor) and *Vitis vinifera* (Vvi).


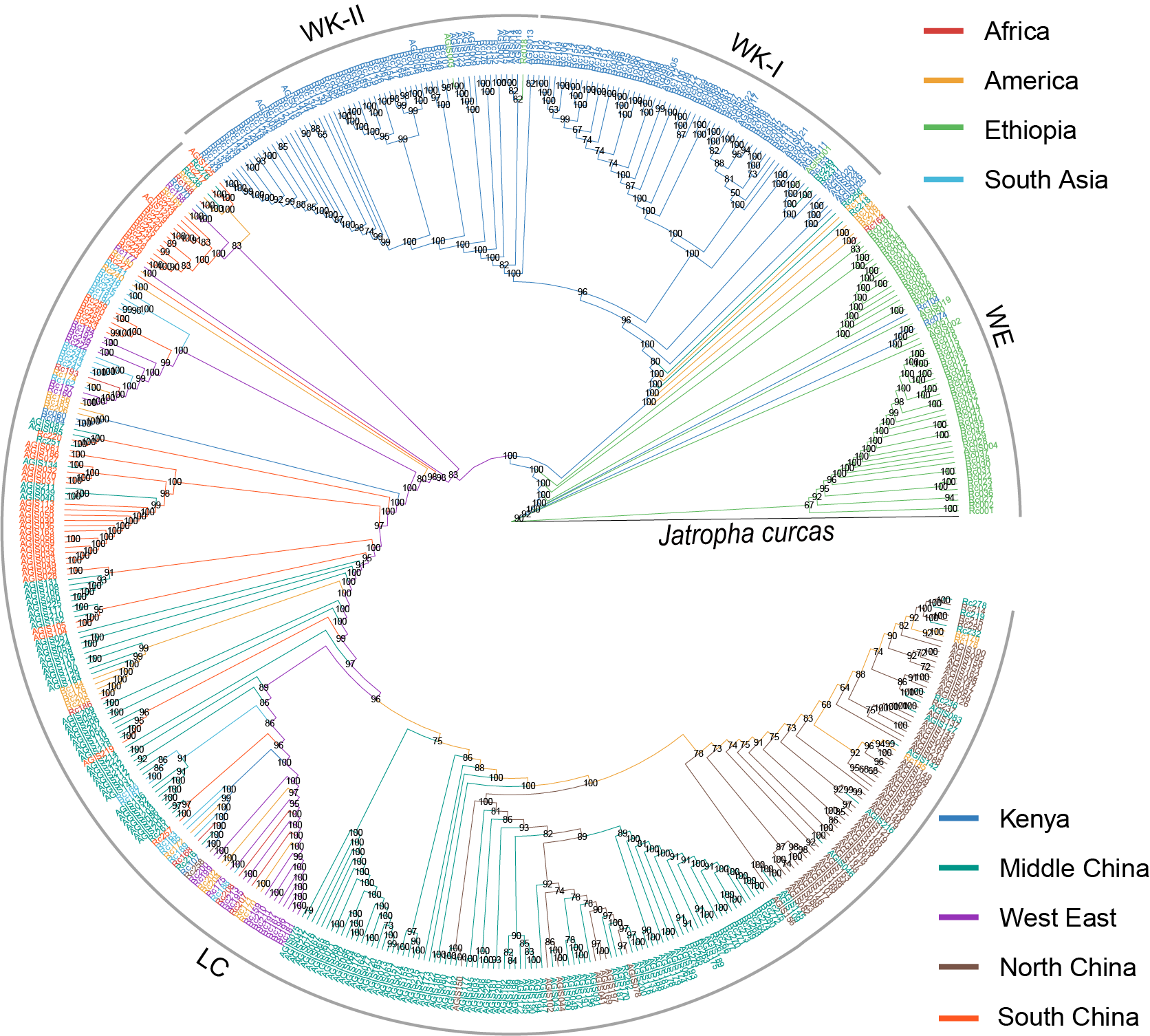


**Figure S6**. A rooted phylogenetic tree of 505 worldwide castor bean accessions based on maximum likelihood with *Jatropha curcas* as outgroup. The number on the clade indicates the value of bootstrap. The colored lines represent the sample source.


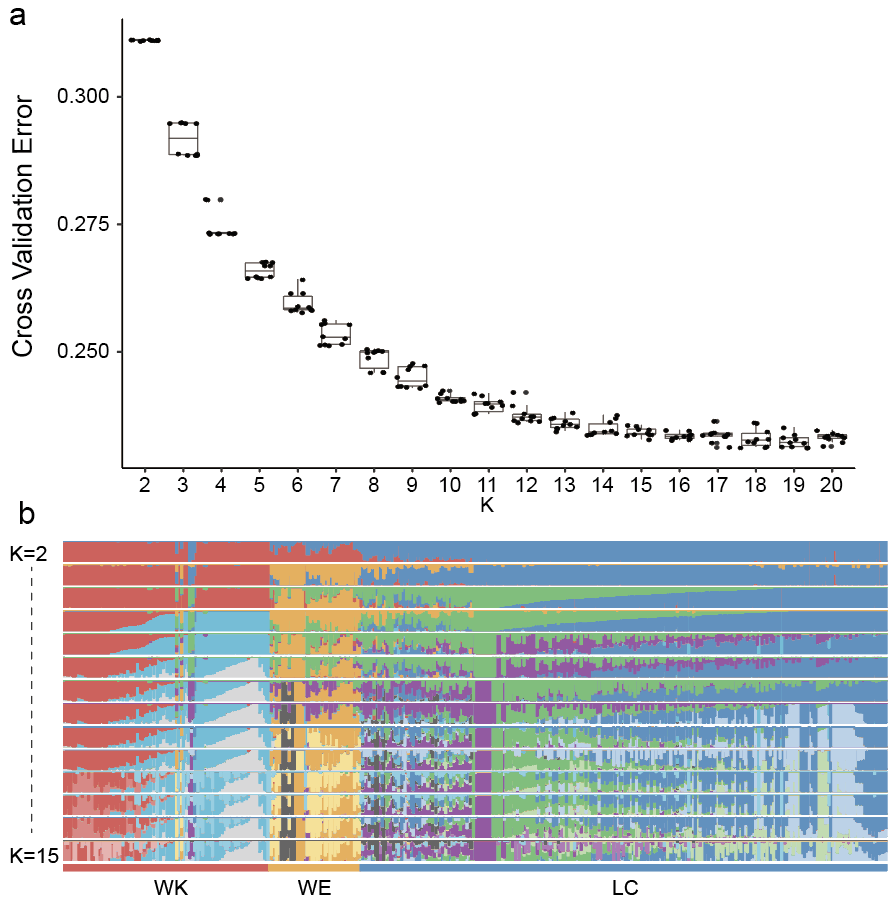


**Figure S7. Population structure analysis in castor bean.** (a) Cross validation error with the change of K values (from 2 to 20). K=10 indicates the optimum group number. (b) ADMIXTURE plots for all accessions supports genetic difference between WE, WK and LC. K values (the number of clusters) from 2 to 15 are shown.

**
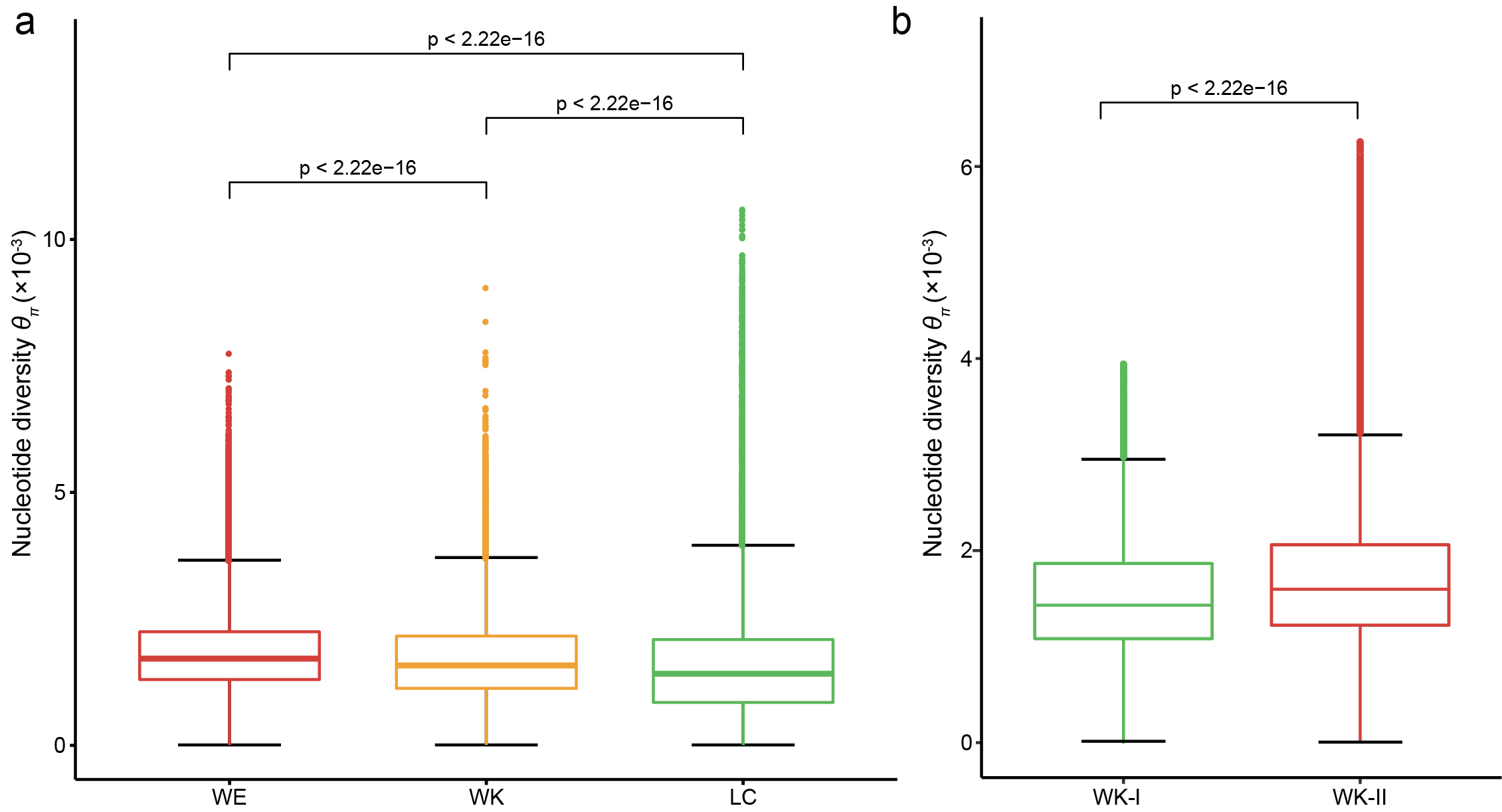
**

**Figure S8. Nucleotide diversity in castor bean populations or subgroups.** (a) Comparison of nucleotide diversity between castor bean populations WE (wild Ethiopia population), WK (wild Kenya population) and LC (landraces and cultivars). (b) Comparison of nucleotide diversity between WK-I and WK-II. Significance was calculated by Kruskal-Wallis test.


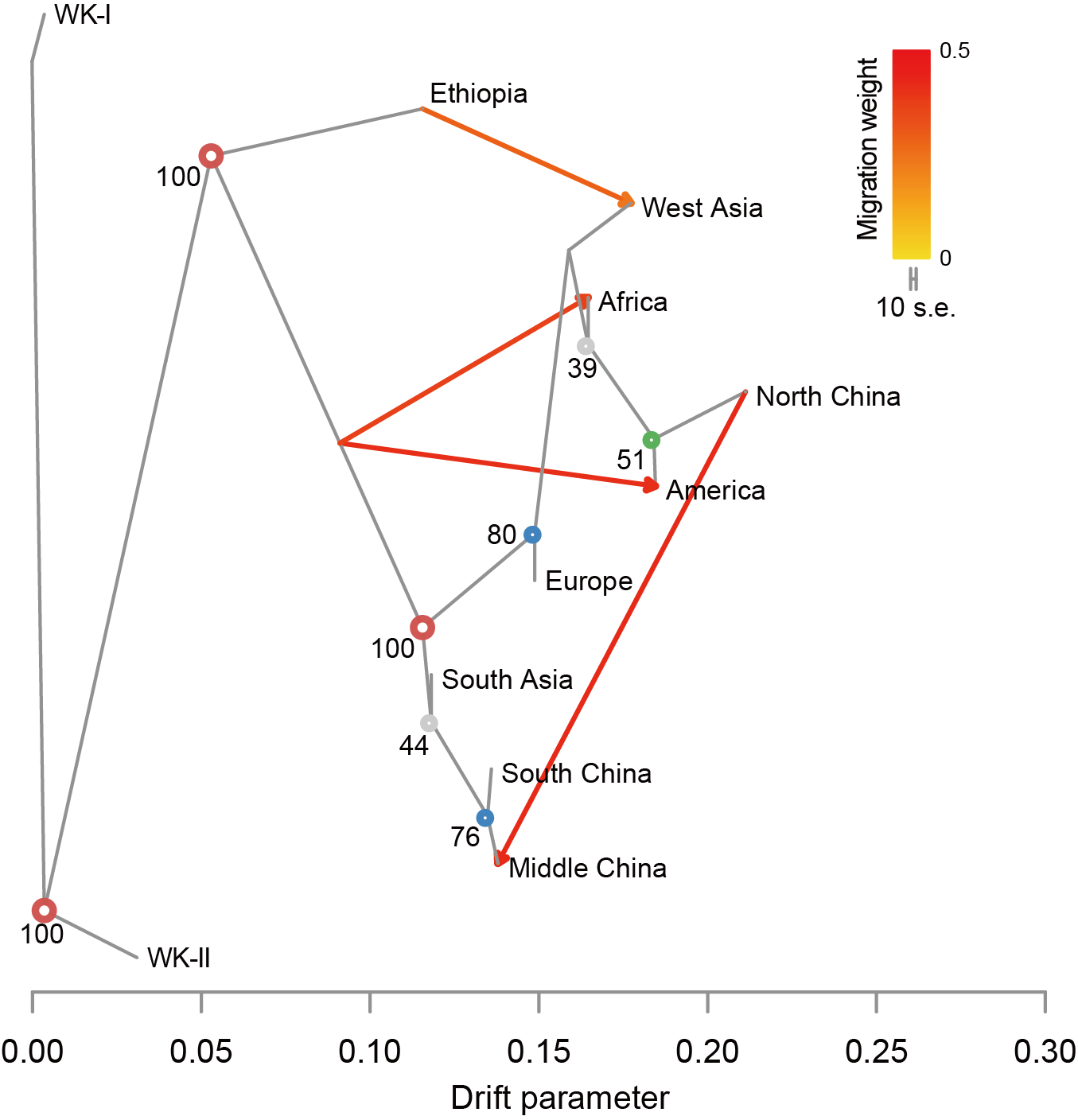


**Figure S9. Inferred population splits and admixture of castor bean using TreeMix**. Bootstrap support values are provided for each node. Lines with arrows indicate the direction of gene flow and the color scale shows the migration weight of gene flow.


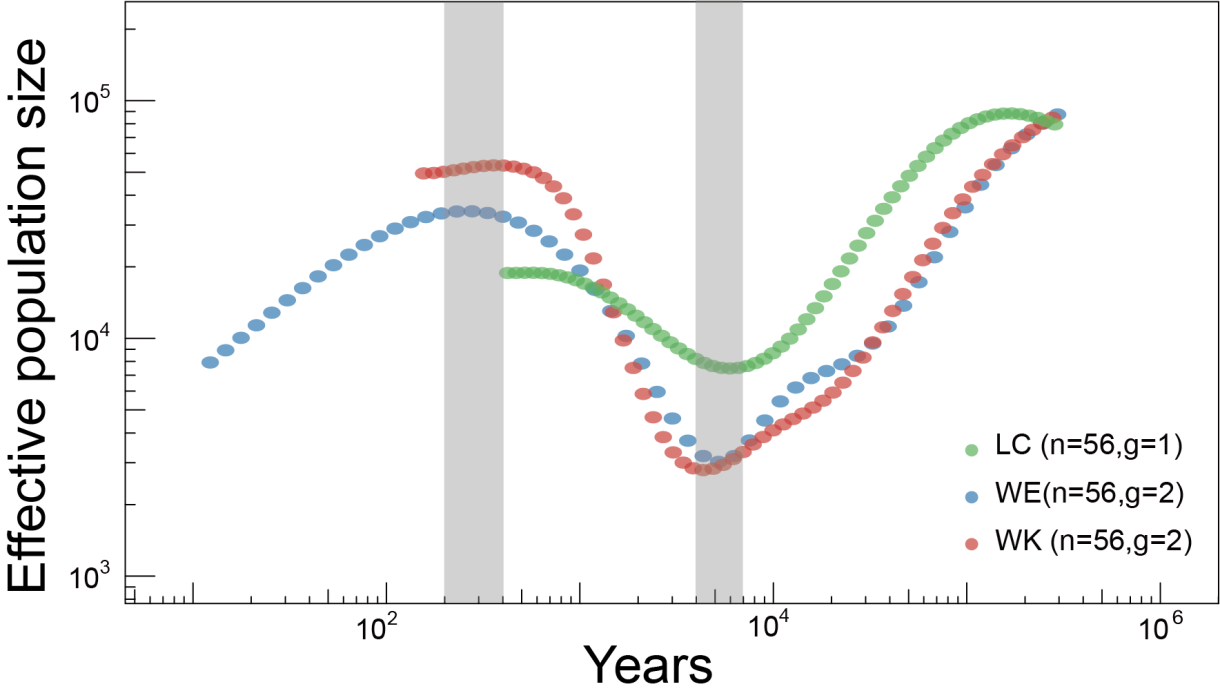


**Figure S10. Effective population size (Ne) inferred by SMC++ based on WGS SNPs data for WE, WK and LC.** A marked decline of Ne in castor bean from 100,000 to ~4400 - 6000 years before present (YBP) is evident with the lowest point highlighted by the grey bar on the right. After this Ne gradually expanded, reaching a maximum ~400-200 years ago (highlighted by the left grey).


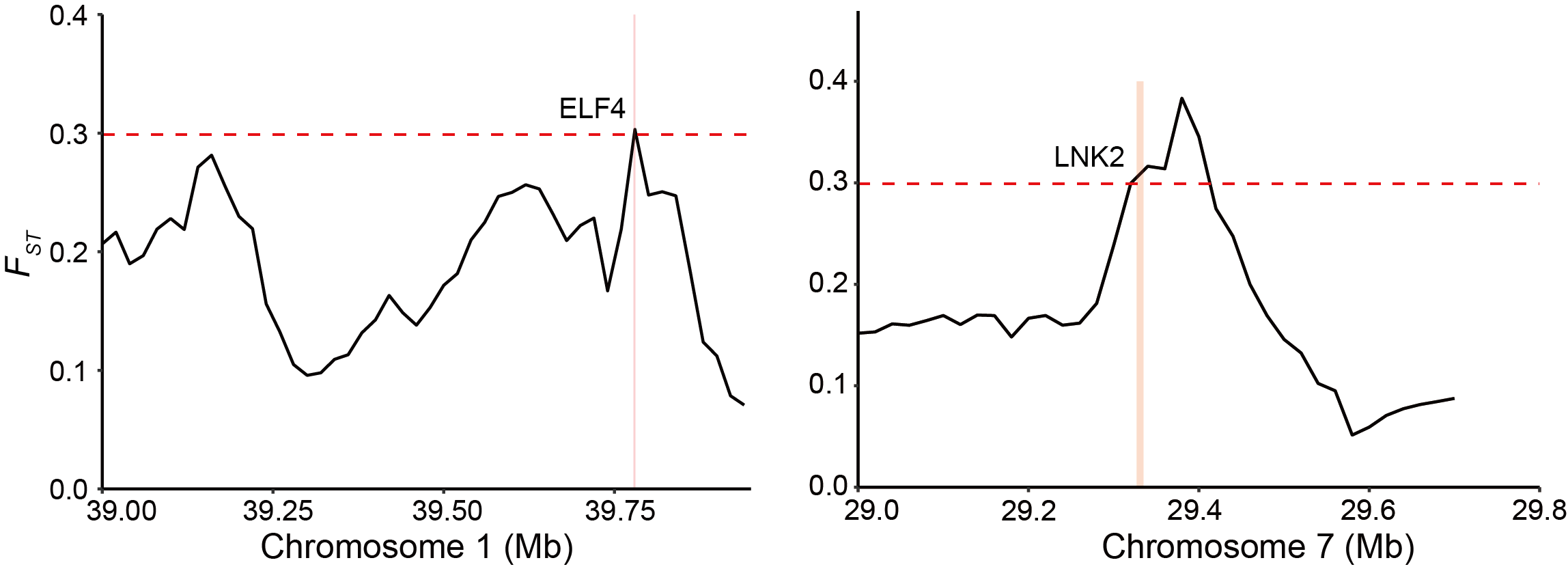


**Figure S11. Two regions of the genome containing the top 5% of *F_ST_* between WK (wild Kenya) and WE (wild Ethiopia) group.** Candidate genes *ELF4* and *LNK2* are indicated. The dashed horizontal line indicates the threshold for top 5% *F_ST_* (0.299).


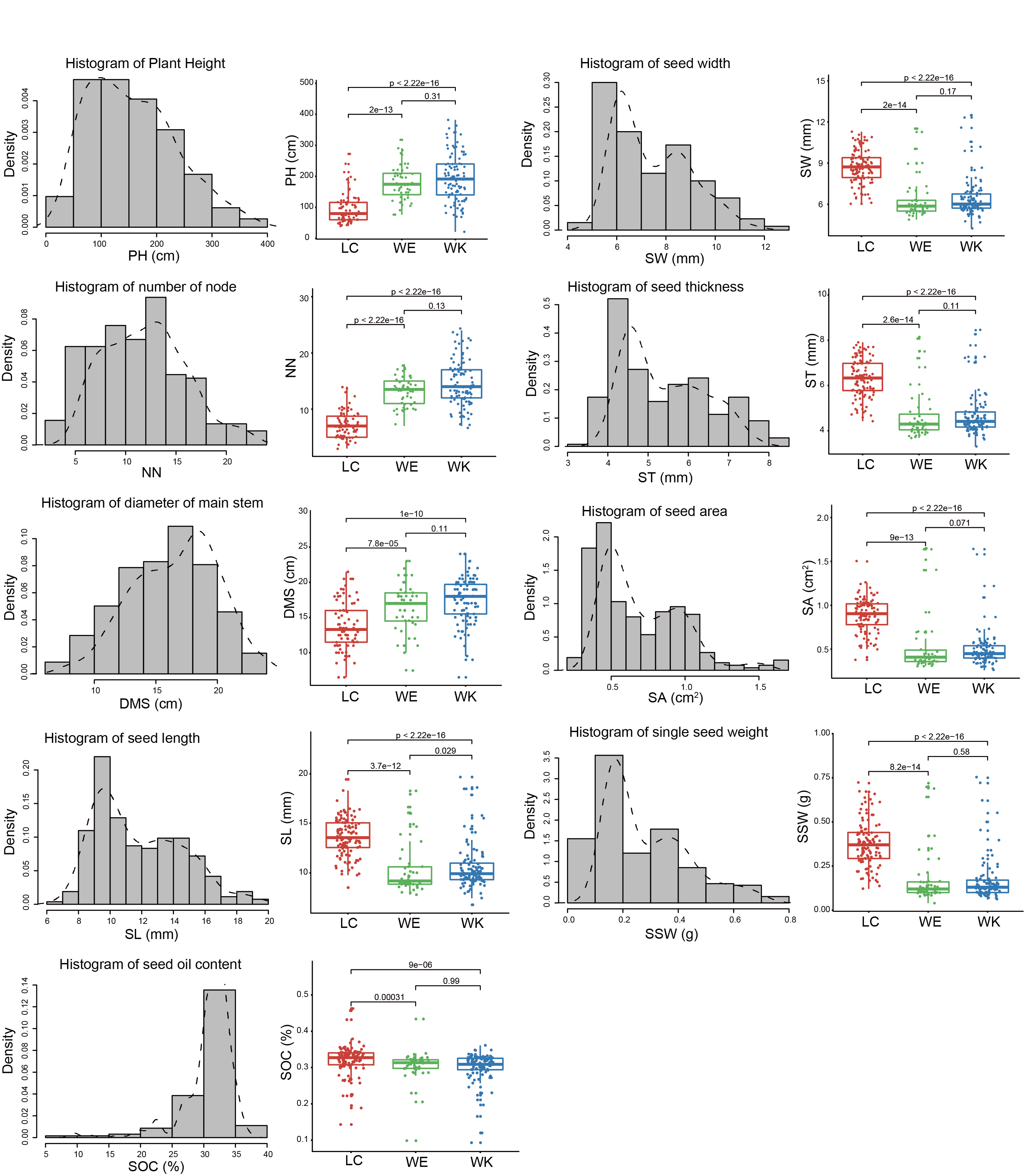


**Figure S12. Histogram and boxplot of nine agricultural traits**. Traits comprise plant height (PH), number of nodes (NN), diameter of main stem (DMS), seed length (SL), seed oil content (SOC), seed width (SW), seed thickness (ST), seed area (SA) and single seed weight (SSW) and represent data from our castor bean phenotyping population. Boxplots indicate phenotypes of the three groups: LC (landrace and cultivar), WE (wild Ethiopia) and WK (wild Kenya) with p-values shown (Wilcoxon test). Same comment as fig S8: What do the boxes, bars etc mean? Means or median? IQR or other range?


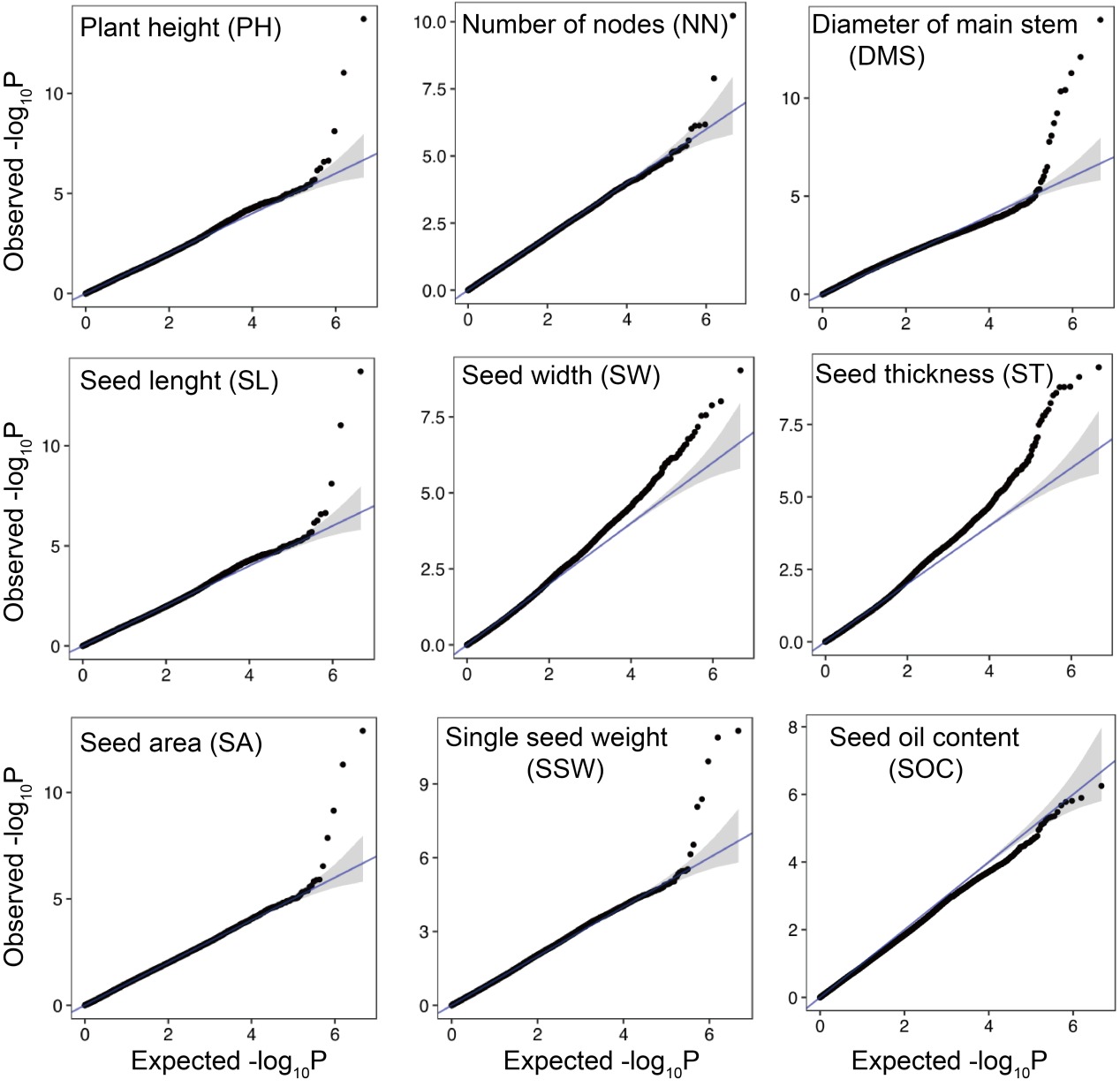


**Figure S13. Quantile-quantile plots for nine agricultural traits by comparing the observed –log_10_P with expected –log_10_P of GWAS.**


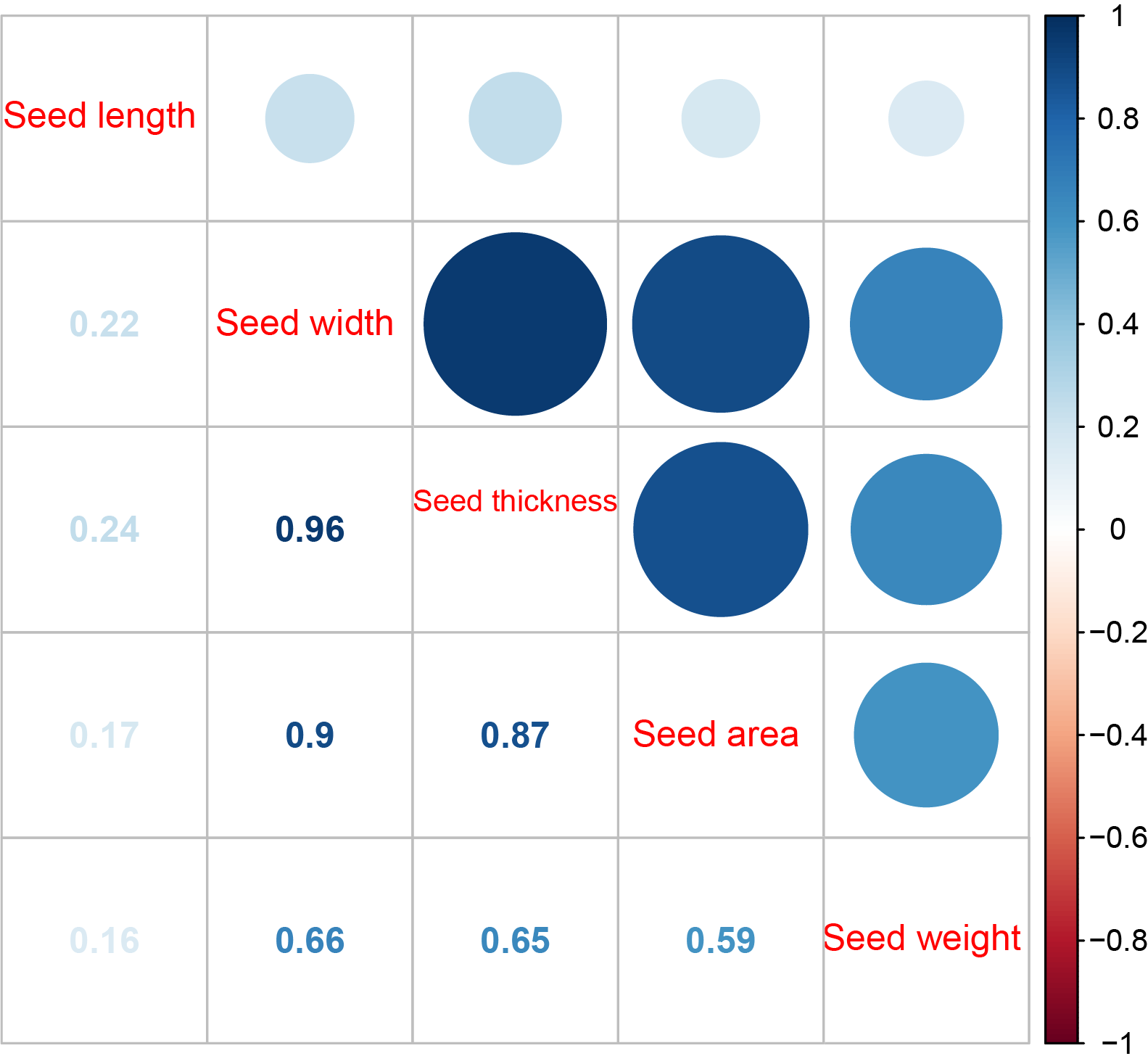


**Figure S14. Correlation of five seed traits,** seed length (SL), width (SW), thickness (ST), area (SA), single seed weight (SSW). The number and color in the grid indicate the Pearson's correlation coefficient.
