# Supplementary material for: Genomic insights into the origin, domestication and genetic basis of agronomic traits of castor bean
Source: Genome Biol. 2021 Apr 20;22:113. doi: 10.1186/s13059-021-02333-y (PMC8056531; doi:10.1186/s13059-021-02333-y)
Supplement: Supplementary file 3 — Additional file 3. Review history. [file 13059_2021_2333_MOESM3_ESM.docx]

Review History

**First round of review**

**Reviewer 1**

**Are you able to assess all statistics in the manuscript, including the appropriateness of statistical tests used?**

**Yes**

**Comments to author:**

In the study titled with "How a tree became an annual crop: genomic insights into the origin, domestication and genetic basis of architecture of castor bean" (GBIO-D-20-01867), Xu et al. have de novo assembled a high-quality genome of wild castor bean and re-sequenced a large diverse natural panel with NGS. The authors have applied these great resources to explore the evolution of Euphorbiaceae, the domestication of a typical non-food plant, and the genetic basis of various castor bean key traits. They made several findings that are really interesting, especially the link between species divergence and the geographic/climate changes.

I believe this study will draw general interest to the community. I would be very happy if following concerns could be further addressed:

1. On the origination and domestication of castor bean:

a. While authors claimed in the introduction that there's still arguing about the domestication center of castor bean, but what it feels in the following is they directly consider the accessions from Ethiopia and Kenya as "domesticated ancestors".

b. I didn't understand why the diversity of West Asia castor beans are comparable to those from East Africa [Line 217-]. The authors only indicated a "center of genetic diversity" in figure 6, but gave no explanation in the main text on this.

c. Based on the two above, I am thus very confused. Is there a possibility that castor beans are domesticated (by human!) from West Asia, and the differences between Ethiopia/Kenya and West Asia are just caused by natural selection, local adaptation, or species/population divergence?

d. Even though we accept the domestication from East Africa, but the descriptions on which one is the domesticated ancestor is also confused, WE or WK or both? While they claimed that "WE is the earliest diverged to domesticated lines" [Line 185], then "WK showed the largest difference, and WE shared the most of genetic component, with cultivars" [Line 194-], and later "Collectively, our results clearly reveal that accessions from Kenya and Ethiopia are the wild progenitors of cultivated castor bean" [Line 224-]. Maybe the authors suggest both the WE and WK are the wild progenitors, but how can it be when these two are already diverged at ~7000 YBP, while domestication occurs at ~3000 YBP?

e. And there are several points that seem very interesting but the authors directly give simple interpretations without any evidence or references, for example: "Seven LC accessions (landraces) were scattered in the wild populations, possibly resulting from a recent introduction directly from the wild population in East Africa" [Line 178]; "...the diversity ratio of Pi_wild/Pi_cultivar in castor bean was quite small...This showed that castor bean may experience a weaker genetic bottleneck and/or that cultivated accessions have repeatedly received gene flow from wild accessions, increasing genetic diversity" [Line 202-]; and "Decay of LD occurred over a substantially shorter distance in wild populations than in domesticated population, correlating with expectations based on greater outcrossing in the wild population" [Line 210-]. I mean, all these findings can be explained by multiple angles, but the interpretations here seem not that straightforward or the most key factor to me.

f. I'm not good at castor bean evolution, but the claim on the direct domesticated ancestor should be made very carefully. At least the authors should have convinced themselves first.

2. On how a perennial and woody tree became an annual semi-woody crop:

Well, this should be another uplifting part of this study, and I believe the authors also expect to explore so since they made this into the title and referred several times in the text. However, after reading the manuscript, what I feel is they only use this to represent the castor bean, not exactly providing something new on this topic. Yes, they identified some genes affecting flowering time or plant architecture that responses to selection, but nothing on how they contribute to lifestyle changes. I personally suggest the authors could reduce this sort of overstatement.

3. Additional confusions or suggestions:

a. Line 63: To me, castor bean produces vital raw materials for many industrial application, I don't understand why the authors put the "bio-weapon" in the most significant position after they claiming the importance of castor bean.

b. Line 70: should be "…dated to ~24,000 years before present…", and the abbreviation of "YBP" is misused as "YPB" in the figure 6.

c. In Fig.3C, the points of WE and WK are too small to be distinguished.

**Reviewer 2**

**Are you able to assess all statistics in the manuscript, including the appropriateness of statistical tests used?**

Yes

**Comments to author:**

In this paper, Xu et al. carried out a genome-wide investigation of castor bean by de novo assembled a reference genome and population analyzed 505 accessions via resequencing. They provided a high quality genome and other necessary annotation information for castor bean. Their study told a story of demographic and domestication of castor bean and detected genetic loci for 3 growth and 5 seed traits by GWAS and QTL mapping of RIL population.

1. In the part of comparative genome analyses, the authors used genomes of 4 species in Euphorbiaceae and Arabidopsis thaliana to conduct orthologous family and expanded/contracted gene analysis (Fig. 1b, c). Except castor bean, all species used in Euphorbiaceae are belong to subfamily Crotonoideae. It is better to add the genome of Mercurialis annua (published in 2019, not too new) into these analysis, which may give a relative complete pattern of gene family within Acalyphoideae, and between Acalyphoideae and Crotonoideae.

2. The 4DTv result shows that R. communis shares similar distance distribution with J. curas, V. fordii and V. vinifera. The authors claimed that M. esculenta and H. brasiliensis "experienced an additional recent whole-genome duplication". However, the 4DTv of M. esculenta and H. brasiliensis doesn't shows a same peak overlapped with R. communis or V. vinifera. Does it supports an "additional" WGD or "new" WGD event? This need to be carefully considered.

3. We know that the Egyptian took usage of costar bean in a quite early time (~3500 years ago). This time is close to the date of putative domestication event the authors claimed (East Africa ca. 3200 years ago). It will be quite interesting to determine if the Egyptian laid a foundation of the domestication of costar? But, looks like the authors didn't took so much samples from Egypt.

4. In addition, the sample from another diversity center seems not enough as well (Arabian Peninsula, Sub continents of India). Selection of representative enough samples is necessary to polish the demographic history and domestication model. I would suggest more representative samples need to be added. Moreover, I am not sure is there any wild costar bean outside of Ethiopia and Kenya. If it has, these wild costar bean accessions have to be added.

5. The WK-I and WK-II were not marked on Figure 2. The WK-I and WK-II show divergence on phylogeny and elevation, but the Fst between WK-I and WK-II (0.08) is not a high value. It will be interesting to know if their genetic structure will become more intense, or be erased by gene flow (I think there's no decisive barrier)? It is better to show the θπby boxplot instead of a single value, so that we can see the degree of significance of θπ between subgroups.

6. I'm not sure whether the GWAS for agricultural trait was conducted by only LC group or all wild and domesticated accessions. Including wild samples may introduce background noise of population structure. In addition, the traits for GWAS from multiple years are better to be estimated by BLUP instead of directly average value.

7. It is interesting that the GWAS signals of several seed traits are located in a same genome region. Are the traits have correlation among each other?

8. One more interesting thing is that costar bean can grow as herb and shrub. Is that change controlled by genetics or environment? Are the growth traits (DMS, NN and PH) stable if planted in different year and/or habitat?

**Reviewer 3**

**Are you able to assess all statistics in the manuscript, including the appropriateness of statistical tests used?**

Yes

**Comments to author:**

The authors de novo assembled a high-quality genome of wild castor bean. They also whole-genome sequenced 505 accessions and identified millions of SNPs. The manuscript provides important genomic resources for the research community of castor bean. The biological insights can be a good contribution as well. I do have a few concerns and suggestions for the authors to address. I hope they will help improve the manuscript.

Major:

1. The manuscript needs better framing. The authors conducted a comprehensive analysis of genome assembly, population structure, demographic history, environmental adaptation, and GWAS. However, these topics are a little bit too broad and lack connections. One way to improve this is to rewrite the introduction. The authors are supposed to show clear motivation and state specific questions or hypothesis. Then the whole manuscript can be better organized based on the questions or hypothesis.

2. If castor bean was domesticated in Ethiopia, how to explain that West Asian castor bean has the greatest diversity (Line 217-218)?

3. Fig. 2 shows population differentiation using unrooted tree, structure, and PCA, but none of them can demonstrate the origin of domestication. I think the rooted tree in the supplementary document are suitable to show the origin, so it needs to be moved to the main figure and modified by adding branch length.

4. Fig. 3 and fig. 6 shows environmental adaptation and migration post-domestication. They are not relevant to the key question in the introduction (Line 84-86). As I mentioned, the manuscript needs restructuring.

5. Can the authors perform some joint analysis of selection scan and GWAS analysis. The Manhattan plots from the two analyses are supposed to have something in common, if the GWAS analysis was on domestication traits.

6. The language needs improvement. For example, US$1,340 should be $1,340 (Line 61). These are many misuses though the manuscript, but I am not listing them one by one. It would be great if the authors can review the manuscript very carefully and find them.

Minor:

1. Line 71, I would not compare the importance of food-crops and non-food crops, because they have different uses.

2. Line 133-135, ribosome, ABC transporters, and pyrimidine are not processes.

3. Line 165-167, there are no true values in either SNPs in this paper or RNA-seq data, so the accuracy cannot be obtained by comparing them.

4. Line 194-195, the statement is not true.

**Authors Response**

**Point-by-point responses to the reviewers’ comments:**

Comment

Reviewer #1:

In the study titled with "How a tree became an annual crop: genomic insights into the origin, domestication and genetic basis of architecture of castor bean" (GBIO-D-2001867), Xu et al. have de novo assembled a high-quality genome of wild castor bean and re-sequenced a large diverse natural panel with NGS. The authors have applied these great resources to explore the evolution of Euphorbiaceae, the domestication of a typical non-food plant, and the genetic basis of various castor bean key traits. They made several findings that are really interesting, especially the link between species divergence and the geographic/climate changes. I believe this study will draw general interest to the community. I would be very happy if following concerns could be further addressed:

1. On the origination and domestication of castor bean: a. While authors claimed in the introduction that there's still arguing about the domestication center of castor bean, but what it feels in the following is they directly consider the accessions from Ethiopia and Kenya as "domesticated ancestors".

*Response: Thank you for your criticism. We have re-organized the introduction by taking this into account. Please see lines 72-95.*

b. I didn't understand why the diversity of West Asia castor beans are comparable to those from East Africa [Line 217-]. The authors only indicated a "center of genetic diversity" in figure 6, but gave no explanation in the main text on this.

*Response: This is a good suggestion. According to previous studies (mainly based on morphological diversity), West Asia has been considered as one of the diversity centers for castor bean (see ref. 9 in the main text). We mentioned this in the previous manuscript (line 77 “… four centers of diversity have been proposed…”). In the section of the manuscript the reviewer commented on, we are presenting our finding that there is a level of genetic diversity in this group of landraces and cultivars that is comparable to that in the wild populations, and substantially higher than the cultivars or landraces from other regions.*

*We explain this finding later in the manuscript and have added more details (see lines 231-243). Briefly, we attribute this to one (or more) of the following reasons: 1) The cultivated accessions in West Asia may have repeatedly received gene flow from wild accessions. To test this hypothesis, we employed TreeMIX to measure gene flow and migration events (see Methods part in lines 723-730) and found, indeed, the direction of gene flow was from Ethiopia to West Asia (Additional file 1: Figure S9); 2) Wild individuals may have previously existed in this area (although germplasm with typical wild phenotype is not reported or found in West Asia) which may have contributed to the high genetic diversity in this region.*

c. Based on the two above, I am thus very confused. Is there a possibility that castor beans are domesticated (by human!) from West Asia, and the differences between Ethiopia/Kenya and West Asia are just caused by natural selection, local adaptation, or species/population divergence?

*Response: This is a good concern. Based on current study the castor bean from West Asia exhibit high genetic diversity, but we cannot say that this was the site of domestication because there is no evidence for wild castor bean in West Asia. We agree with you on that there could have been wild-like group in the past ~6000 years before present (see ref 10 in this study) from which the West Asian LC group may be derived by natural selection, local adaptation and domestication. Currently, we cannot specifically determine the location of domestication yet, but we can confidently point out that the East African accessions are the closest extant wild relatives of domesticated castor bean. We have edited the language throughout to reflect this (see lines 248-2543 and 487-500).*

d. Even though we accept the domestication from East Africa, but the descriptions on which one is the domesticated ancestor is also confused, WE or WK or both? While they claimed that "WE is the earliest diverged to domesticated lines" [Line 185], then "WK showed the largest difference, and WE shared the most of genetic component, with cultivars" [Line 194-], and later "Collectively, our results clearly reveal that accessions from Kenya and Ethiopia are the wild progenitors of cultivated castor bean" [Line 224-]. Maybe the authors suggest both the WE and WK are the wild progenitors, but how can it be when these two are already diverged at ~7000 YBP, while domestication occurs at ~3000 YBP?

*Response*: *This section was not clear enough in the previous manuscript and we have rephrased it to improve the clarity. In this revised version we made it clear that both WE and WK accessions are the extant wild progenitors. As mentioned above, before the genetic differentiation between wild WE and WK, vast areas of the natural spreading of castor bean had been uncovered in the prehistoric age, as revealed by anthropological records in South Africa, Suda and Iraq. It is possible that the early stage of domestication of castor bean by harvesting the wild progenitors from East Africa or by selecting and managing local wild-like accessions. We have reorganized this part and made it clearer accordingly (see lines 248-2543 and 487-500).*

e. And there are several points that seem very interesting but the authors directly give simple interpretations without any evidence or references, for example: [1] "Seven LC accessions (landraces) were scattered in the wild populations, possibly resulting from a recent introduction directly from the wild population in East Africa" [Line 178];

*Response: The seven accessions (landraces) clustered in the wild population are indeed interesting, however, we have limited information regarding their origins. The passport data only indicate that they are from the USA which we assume to mean that they were collected from East Africa and have been grown (or even bred) in the USA. We rephrased this sentence, ‘possibly resulting from a recent introduction from East Africa or have resulted from recent breeding’ (see lines 195-198).*

f. I'm not good at castor bean evolution, but the claim on the direct domesticated ancestor should be made very carefully. At least the authors should have convinced themselves first.

*Response*

*Thank you for your criticism and we think that it is constructive. We have checked our wording again and revised it throughout the manuscript to make a more balanced conclusion (see revised Abstract, Discussion and Conclusions sections).*

[2] "...the diversity ratio of Pi_wild/Pi_cultivar in castor bean was quite small...This showed that castor bean may experience a weaker genetic bottleneck and/or that cultivated accessions have repeatedly received gene flow from wild accessions, increasing genetic diversity" [Line 202-];

*Response: This is a good suggestion. We indicate that this ratio suggests that the domestication bottleneck is relatively weak in castor bean, or that an alternative interpretation is that post-domestication gene flow has caused a relative increase in genetic diversity of the domesticated castor bean. Overall, we find some evidence that there is genetic admixture of some landrace accessions (see structure plot figure 2d where there is some shared genetic component between LC and WE at lower values of K) and there is gene flow from wild population to LC population (as mentioned above). Therefore, we have rephrased the text to reflect this, and highlighted the likelihood that this suggests a weak genetic bottleneck (see lines 212-219).*

[3] "Decay of LD occurred over a substantially shorter distance in wild populations than in domesticated population, correlating with expectations based on greater outcrossing in the wild population" [Line 210-]. I mean, all these findings can be explained by multiple angles, but the interpretations here seem not that straightforward or the most key factor to me.

*Response: It has been reported in other crops that LD breaks down faster in the wild relatives, and this is what we believe as the most likely interpretation. We have added a reference (ref. 14 in this study) in this part in support of our viewpoint (see line 225226).*

2. On how a perennial and woody tree became an annual semi-woody crop: Well, this should be another uplifting part of this study, and I believe the authors also expect to explore so since they made this into the title and referred several times in the text. However, after reading the manuscript, what I feel is they only use this to represent the castor bean, not exactly providing something new on this topic. Yes, they identified some genes affecting flowering time or plant architecture that responses to selection, but nothing on how they contribute to lifestyle changes. I personally suggest the authors could reduce this sort of overstatement.

*Response*

*After careful reconsideration, we generally accept your advice. Whilst we do focus on candidate genes that could have changed the overall plant architecture, we are not addressing this question alone. Consequently, we have checked our wording and revised it throughout the manuscript (see revised Title, Discussion and Conclusions sections).*

3. Additional confusions or suggestions:

a. Line 63: To me, castor bean produces vital raw materials for many industrial application, I don't understand why the authors put the "bio-weapon" in the most significant position after they claiming the importance of castor bean.

*Response*

*We have tried to provide a balanced discussion of the uses for castor bean, and we have focused on the more positive uses, and revised this sentence subsequently (see lines 55-58).*

b. Line 70: should be "…dated to ~24,000 years before present…", and the abbreviation of "YBP" is misused as "YPB" in the figure 6.

*Response*

*We have revised these.*

c. In Fig.3C, the points of WE and WK are too small to be distinguished.

*Response: We have revised this.*

Reviewer #2:

In this paper, Xu et al. carried out a genome-wide investigation of castor bean by de novo assembled a reference genome and population analyzed 505 accessions via resequencing. They provided a high quality genome and other necessary annotation information for castor bean. Their study told a story of demographic and domestication of castor bean and detected genetic loci for 3 growth and 5 seed traits by GWAS and QTL mapping of RIL population.

1. In the part of comparative genome analyses, the authors used genomes of 4 species in Euphorbiaceae and Arabidopsis thaliana to conduct orthologous family and expanded/contracted gene analysis (Fig. 1b, c). Except castor bean, all species used in Euphorbiaceae are belong to subfamily Crotonoideae. It is better to add the genome of Mercurialis annua (published in 2019, not too new) into these analysis, which may give a relative complete pattern of gene family within Acalyphoideae, and between Acalyphoideae and Crotonoideae.

*Response: Thank you for pointing this out. In the revised manuscript, we have made an effort to add the genome of Mercurialis annua to our analysis and revised our manuscript accordingly (Figure 1, Table S5 and S6, main text (see lines 135-149) and Methods (see line 643).*

2. The 4DTv result shows that R. communis shares similar distance distribution with J. curas, V. fordii and V. vinifera. The authors claimed that M. esculenta and H. brasiliensis "experienced an additional recent whole-genome duplication". However, the 4DTv of M. esculenta and H. brasiliensis doesn't shows a same peak overlapped with R. communis or V. vinifera. Does it supports an "additional" WGD or "new" WGD event? This need to be carefully considered.

*Response: The recent WGD event that occurred in M. esculenta and H. brasiliensis genome has been reported in previous studies in rubber tree (see ref PMID: 27255837 and figure below) and tung tree (see ref PMID: 32224189), respectively. In our modified analysis we have identified two peaks in M. esculenta and H. brasiliensis and only one in castor bean. Please see the revised Figure 1d.*

3. We know that the Egyptian took usage of costar bean in a quite early time (~3500 years ago). This time is close to the date of putative domestication event the authors claimed (East Africa ca. 3200 years ago). It will be quite interesting to determine if the Egyptian laid a foundation of the domestication of costar? But, looks like the authors didn't took so much samples from Egypt.

*Response: This is a good suggestion. Unfortunately we cannot identify whether the seeds in the ancient tomb were harvested from wild or domesticated castor bean, or some intermediate. More samples from Egypt would indeed provide some insights into the domestication of castor bean, but there are no records of wild castor bean from Egypt and only very few cultivars or landraces are available. In addition to our work, previous studies have also shown limited geographic genetic structure in landrace and cultivated castor bean including accessions from Egypt (see ref. 13-15 in this study), therefore it does not appear that any Egyptian accessions show a distinct hallmark of being the earliest domesticates. We have added more about the importance of North African castor bean (especially Egypt and Sudan) during castor bean domestication (see lines 487-500).*

4. In addition, the sample from another diversity center seems not enough as well (Arabian Peninsula, Sub continents of India). Selection of representative enough samples is necessary to polish the demographic history and domestication model. I would suggest more representative samples need to be added. Moreover, I am not sure is there any wild costar bean outside of Ethiopia and Kenya. If it has, these wild costar bean accessions have to be added.

*Response: As mentioned above, wild castor bean is restricted to the areas (Ethiopia and Kenya) we sampled, but we acknowledge that further accessions of landraces from key locations (especially Northern Africa) would be ideal but are not available. We have added this point to our discussion (see lines 487-500). Cultivars in the India subcontinent is less critical and has been well studied, and some studies considered that castor bean in Arabian Peninsula was introduced from India (see ref 8 in this study). Overall, our current samples have covered the core collection of castor bean germplasms worldwide as estimated in previous study (ref: PMID: 28094539). Thus, we think that our study of 505 samples is generally sufficient to dissect genetic differentiation during castor bean domestication and accurately discern the demographic history.*

5. The WK-I and WK-II were not marked on Figure 2. The WK-I and WK-II show divergence on phylogeny and elevation, but the Fst between WK-I and WK-II (0.08) is not a high value. It will be interesting to know if their genetic structure will become more intense, or be erased by gene flow (I think there's no decisive barrier)? It is better to show the θπ by boxplot instead of a single value, so that we can see the degree of significance of θπ between subgroups.

*Response: Sorry, we have marked WK-I and WK-II on Figure S6 by now. Although we observed low differentiation, there are clearly two groups based on the phylogenetic analysis, and groups WK-I and WK-II are distributed on both sides of the Kenyan Rift (figure 3d) but in very close proximity. Indeed, it* *remains to be seen if this is to be eroded or maintained over time because of adaptation (potentially related to altitude). We have revised this part (see lines 300-305) and provided an additional boxplot to show θπ (see Additional file 1: Figure S8b).*

6. I'm not sure whether the GWAS for agricultural trait was conducted by only LC group or all wild and domesticated accessions. Including wild samples may introduce background noise of population structure. In addition, the traits for GWAS from multiple years are better to be estimated by BLUP instead of directly average value.

*Response: This is a reasonable consideration. To reduce the effect of population structure we preformed the necessary controls, for example using PCA values and kinship (K) matrices, as described in Methods section in the previous version (see lines 792-794). We agree that using data from multiple years would be better, but wild castor bean flowers infrequently and we could not have data for two years for wild samples. In addition, note that the traits we measured were consistent between the years (see also response below). Combining the years matches the approach taken by another study on castor bean (ref. 16) and we note that several GWAS signals we recorded had been seen previously, suggesting that our approach did not significantly affect our effort to accurately identify GWAS signals.*

7. It is interesting that the GWAS signals of several seed traits are located in a same genome region. Are the traits have correlation among each other?

*Response: This is a good point. This is true that there appears to be a correlation between some of these traits, hence we expect some traits to show overlapping GWAS signals (see additional file 1: Figure S14). We also added more explanation about this issue (see lines 426-428).*

8. One more interesting thing is that costar bean can grow as herb and shrub. Is that change controlled by genetics or environment? Are the growth traits (DMS, NN and PH) stable if planted in different year and/or habitat?

*Response: Based on our field observation and our controlled experiments, the wild castor bean always exhibits a perennial and woody tree phenotype with an elongated stem while the cultivated castor bean is annual, semi-woody crops, suggesting that the major differences in growth habit are stable. Differences between years are evident in some traits, however the three architecture traits used in this study (DMS, NN and PH) were stable.*

Reviewer #3: Comments for the manuscript entitled "How a tree became an annual crop: genomic insights into the origin, domestication and genetic basis of architecture of castor bean". The authors de novo assembled a high-quality genome of wild castor bean. They also whole-genome sequenced 505 accessions and identified millions of SNPs. The manuscript provides important genomic resources for the research community of castor bean. The biological insights can be a good contribution as well. I do have a few concerns and suggestions for the authors to address. I hope they will help improve the manuscript.

Major:

1. The manuscript needs better framing. The authors conducted a comprehensive analysis of genome assembly, population structure, demographic history, environmental adaptation, and GWAS. However, these topics are a little bit too broad and lack connections. One way to improve this is to rewrite the introduction. The authors are supposed to show clear motivation and state specific questions or hypothesis. Then the whole manuscript can be better organized based on the questions or hypothesis.

*Response: Thank you for your constructive suggestion. We have reorganized and rewritten several parts of the manuscript to better present our results.*

2. If castor bean was domesticated in Ethiopia, how to explain that West Asian castor bean has the greatest diversity (Line 217-218)?

*Response: This is a good concern. West Asian castor bean has the greatest genetic diversity of the landraces and cultivars. Reviewer 1 also commented on this and we have addressed it throughout the manuscript to make a more balanced interpretation (see details above).*

3.Fig. 2 shows population differentiation using unrooted tree, structure, and PCA, but none of them can demonstrate the origin of domestication. I think the rooted tree in the supplementary document are suitable to show the origin, so it needs to be moved to the main figure and modified by adding branch length.

*Response: This is a good suggestion. We have moved the rooted tree in the Figure 2, however, we have not modified the branch lengths because this causes loss of resolution and it is harder to see some of the relationships.*

4. Fig. 3 and fig. 6 shows environmental adaptation and migration post-domestication. They are not relevant to the key question in the introduction (Line 84-86). As I mentioned, the manuscript needs restructuring.

*Response: As mentioned above we have rewritten the introduction section following your suggestions (see lines 72-103).*

5. Can the authors perform some joint analysis of selection scan and GWAS analysis. The Manhattan plots from the two analyses are supposed to have something in common, if the GWAS analysis was on domestication traits.

*Response: This is an important point. Actually, we have performed joint analysis of selection scan and GWAS analysis in the previous manuscript, and please see the figures 4 and 5 and Table S16.*

6. The language needs improvement. For example, US$1,340 should be $1,340 (Line 61). These are many misuses though the manuscript, but I am not listing them one by one. It would be great if the authors can review the manuscript very carefully and find them.

*Response: We have smoothed the English carefully.*

Minor:

1. Line 71, I would not compare the importance of food-crops and non-food crops, because they have different uses.

*Response: This is a good suggestion. We have revised it accordingly (see lines 65-66).*

2. Line 133-135, ribosome, ABC transporters, and pyrimidine are not processes.

*Response: Thank you for pointing this out. We have revised it (see lines 140-145).*

3. Line 165-167, there are no true values in either SNPs in this paper or RNA-seq data, so the accuracy cannot be obtained by comparing them.

*Response: We have added the true SNP number identified in RNA-seq data which now highlights that >98% of SNPs were detected in both analyses (see lines 175- 179).*

4. Line 194-195, the statement is not true.

*Response: We have removed this statement.*

*Overall, we thank the reviewers for their insightful comments and hope that our revisions have removed any concerns over the data or its interpretation.*

**Second round of review**

**Reviewer 1**

I really appreciate that the authors have put their huge efforts into the revised manuscript, the genome assembly and the GWAS stuff are solid to me. However, most of the confusion about castor bean origination and domestication still persists. I fully understand we may not know what exactly happened during the past, but only change the previous affirmative statements into euphemistic ones (with so many "may" "possible" stuffs) may not indeed help the manuscript improving. Beyond the confirmation of previously evolutionary propositions and several related genes (inferred based on homologous genes without any validations) being identified within selective sweeps, it's still not clear what general "novel insights into the understanding of genomic evolution in the Euphorbiaceae" have been provided in the present study.

**Reviewer 2**

I do not have more comments. Only the title is too abroad, it is better to delete "From a tree to an annual crop:".

**Reviewer 3**

All my questions have been well addressed in this revision. By providing more convincing evidence and reasonable inference, the authors showed a much clearer domestication process of castor bean. It is great to see some common signals from the joint analysis of selective sweeps and GWAS. Also, the readability of the manuscript has been improved substantially. This is a good revision and I do not have other questions for the manuscript.

**Authors Response**

**Point-by-point responses to the reviewers’ comments:**

Reviewer #1: I really appreciate that the authors have put their huge efforts into the revised manuscript, the genome assembly and the GWAS stuff are solid to me. However, most of the confusion about castor bean origination and domestication still persists. I fully understand we may not know what exactly happened during the past, but only change the previous affirmative statements into euphemistic ones (with so many “may” “possible” stuffs) may not indeed help the manuscript improving. Beyond the confirmation of previously evolutionary propositions and several related genes (inferred based on homologous genes without any validations) being identified within selective sweeps, it’s still not clear what general “novel insights into the understanding of genomic evolution in the Euphorbiaceae” have been provided in the present study.

*Response: We carefully consider your criticism of the manuscript, especially the origin and domestication in castor bean. Many factors such as extensive and continued introduction in many regions, predominant outcrossing under natural conditions and its widespread escape from cultivation, complicate the study on the origin and domestication in castor bean. In the previous version, we used some vague language, which did not do justice to some of our findings, therefore in the revised version we have strengthened the language in places to reflect this.*

*In addition, we have removed this overstated sentence “novel insights into the understanding of genomic evolution in the Euphorbiaceae”.*

Reviewer #2: I do not have more comments. Only the title is too abroad, it is better to delete "From a tree to an annual crop:".

*Response: We have revised the title according to your suggestion.*
